# Digitally Assisted Training System for Home-Based Scoliosis Therapy: Proof-of-Concept and Technical Verification Study

**DOI:** 10.2196/93721

**Published:** 2026-10-29

**Authors:** Florian Günther, Sandra Hunger, Michael Werner, Nikolaus Landerer, Christoph-Eckhard Heyde, Welf-Guntram Drossel

**Affiliations:** 1 Functionalization Technologies, Fraunhofer Institute for Machine Tools and Forming Technology (IWU), Nöthnitzer Straße 44, Dresden, 01187, Germany, 49 351 4772-2160; 2 Department of Orthopaedic, Hospital of Orthopaedic Surgery, Traumatology and Plastic Surgery, University Hospital Leipzig, Leipzig, Germany; 3 Acoustical Engineering, Fraunhofer Institute for Machine Tools and Forming Technology (IWU), Dresden, Germany; 4 Fuzz Tech IT Solutions GmbH, Leipzig, Germany; 5 Professorship Adaptronics and Lightweight Design, Chemnitz University of Technology, Chemnitz, Germany; 6 Scientific Field Functional and System Integration, Fraunhofer Institute for Machine Tools and Forming Technology (IWU), Dresden, Germany

**Keywords:** scoliosis, Schroth therapy, physiotherapeutic scoliosis-specific exercises, PSSE, training device, exercise system, assistive devices, test bench, spine model, breathing simulation, motion tracking

## Abstract

**Background:**

Schroth therapy is one of the conservative methods used to treat scoliosis. It includes not only therapeutic exercises in physiotherapy facilities but also concepts for independent training at home. Consistent and correct performance of this training has a major impact on the success of the therapy. However, without guidance and supervision by a therapist, patient motivation may decline. In addition, incorrect training techniques may be used, which can have a negative impact on the success of the therapy.

**Objective:**

This study aimed to develop a digitally assisted training system for home-based scoliosis therapy and to verify it technically on a laboratory scale.

**Methods:**

On the basis of training devices used in Schroth therapy, such as mirrors, balance boards, wall bars, and exercise sticks, we developed a concept for a modular, digitally assisted scoliosis training system (iScoolio). The development process included a requirements specification, iterative design drafts, and the transfer of the concepts into a functional demonstrator. Finally, a theoretical evaluation of the use cases and a technical verification of the measurement functions were carried out using specifically developed test scenarios.

**Results:**

A demonstrator with tracking, training, and modification functions was developed, consisting of 4 modules. Technical verification of the integrated measurement functions showed that the angle measurement in motion tracking (n=50) had a mean deviation of 2.29° (SD 1.66°) compared to the ARAMIS 3D optical reference system. Respiration tracking enabled the detection of all simulated respiratory cycles (n=40) and the significant distinction between shallow and deep breathing (*t*_39_=−27.4; *P*<.001). The investigation of the 2 force sensor concepts in the handles (*χ*²_2_=80.0; *P*<.001) and supporting feet (*χ*²_2_=50.0; *P*<.001) showed a significant differentiation between 3 force levels, but approximately linear drift behavior in each case. When tracking the stance width (n=50), the mean value was 15.4 (SD 0.54) cm. Verification of the center of pressure measurement function (n=50) showed that 48% of the measurements had a deviation of ≤10 mm, and 82% of the measurements had a deviation of ≤20 mm.

**Conclusions:**

In this paper, we have presented the iScoolio concept, which has the potential to support home-based scoliosis therapy through adaptable devices and tracking elements. Integrated sensors enable monitoring of movements, breathing, forces, and pressure distribution during training. An associated web application visualizes the recorded data and serves as a guide for performing the exercises. Technical verification indicates that the integrated tracking methods are functional; however, there is still room for optimization in terms of hardware and software. Further validation is required before conducting clinical trials under real-world conditions.

## Introduction

### Background

In the field of conservative scoliosis therapy, therapeutically guided physiotherapeutic scoliosis-specific exercises (PSSE) are supplemented by training units that patients perform independently at home. One of the 7 major schools in the field of PSSE is Schroth therapy, which has established itself worldwide and especially in Germany. Developed in 1920 by Katharina Schroth, this therapeutic method follows five principles: (1) auto-elongation (detorsion), (2) deflection, (3) derotation, (4) rotational angular breathing (RAB), and (5) stabilization. On the basis of diagnostic findings, Schroth also divides the human body conceptually into a 3- or 4-block model (Appendix Chapter 1, Multimedia Appendix 1), which then serves as the basis for the therapy plan. The change in shape of these blocks from a rectangle to a trapezoid and the horizontal positioning visualize the scoliotic trunk deformation and lateral displacement [1].

Another special feature of Schroth therapy compared to other PSSE is RAB. This uses the connection between the ribs and spine and the increase in volume of the chest during breathing to correct the concave and convex areas of the torso through targeted contraction of the torso muscles. This means that every breath can also be a corrective exercise [1]. Some of the frequently used exercises in Schroth therapy, which involve RAB, are “Between Two Poles” (Zwischen zwei Stäben), “50X” (50X Pezziball), and “Sail” (Segel) [1,2]. Further information on these exercises can be found in Multimedia Appendix 2.

These exercises require patients to be able to understand the complex movements cognitively and motorically. This often requires intensive therapy assistance from a therapist or physician, which is not available in the home setting [3]. The lack of safety when training independently and the lack of suitable therapy equipment therefore often lead to reduced motivation, which in turn has a negative effect on the success of the therapy. Various possible solutions are described in the literature: Portable textile systems such as the “Wearable Sensor Array” by Caviedes et al [4], the “Scoliosis Rehabilitation Assistant” by Li et al [5], the “SpineCurer” by Mai et al [6], or the “Wearable Scoliosis Monitoring System” [7] use stretch or inertial sensors placed on the back to track movement. Pattern recognition and linked apps can then be used to monitor scoliosis exercises and provide training instructions [4-6]. Stationary training systems such as the “DISC4SPINE-System” [8,9] or the “rehabilitation exercise system” enable exercises in a seated position [10] or while standing and monitoring of therapy progress using various sensors [8,9]. In addition, several apps and web-based systems are presented, such as “ScolioTrack,” “ScolioMeter,” “ScolioDetector,” “Scoliosis Tracker,” or the “Scoliosis Manager,” which determine the progression of spinal curvature via smartphone, remind patients of appointments, or assist in the management of scoliosis rehabilitation [11,12].

All these wearables, training devices, and apps offer approaches and partial solutions for improving and supporting home-based scoliosis therapy. However, the current literature has not yet identified a holistic system that enables a wide range of exercises and guides and monitors them and, at the same time, increases motivation for training.

### Study Aims

In a previous study [13], we investigated the potential for optimizing home-based scoliosis therapy by conducting online surveys of patients with scoliosis and therapists. The results showed that, according to their own statements, patients exercise significantly less at home than what their therapists recommend. The reasons for this are a lack of motivation and uncertainty when performing complex exercises, a lack of awareness of the importance of training, and the absence of supervision by therapists [14,15].

The patients surveyed stated that their motivation could be increased by training together with friends and acquaintances, by a supportive therapy system, and by a digital profile. We also asked about the popularity of various training devices. It turned out that balance boards, wall bars, mirrors, and exercise sticks are among the most popular devices, but that patients use them relatively rarely at home (≤55.6%). Regarding digital tools, patients attached great importance to an app, video support, and vibration feedback. Therapists saw particular advantages in increasing motivation, improving home-based therapy, monitoring therapy progress, and communicating therapy instructions [13].

On the basis of the results of our survey and the lack of devices on the market, the aim of this study was to develop a novel concept for a holistic and digitally assisted scoliosis training system. Consequently, this study focuses on the technical verification of the demonstrator, serving as a prerequisite for future clinical evaluation and formal medical device development. To this end, we have formulated the following hypotheses:

- H_0_: The integrated sensor framework of the new home-based scoliosis therapy concept provides valid and accurate measurements against technical references.
- H_1_ (motion tracking): The demonstrator yields motion measurements that closely align with high-precision optical reference data across various test bench positions.
- H_2_ (respiration tracking): The demonstrator accurately detects simulated breathing cycles and variations in breathing depth generated by a test bench.
- H_3_ (force tracking): The demonstrator provides clear, load-proportional raw sensor deflections in response to applied forces, confirming its technical capability to capture force variations.
- H_4_ (stance width and center of pressure tracking): The demonstrator reliably determines stance width and center of pressure (COP) using 2 test weights to simulate a 2-foot stance.

## Methods

### Study Design

This study was designed as a proof-of-concept and technical verification study. It describes the development process and the resulting demonstrator, its functionalities using exemplary Schroth exercises, and the technical verification of its measurement functions. The study’s findings correspond to Technological Readiness Level 4.

### Ethical Considerations

As part of the study, no human participants or animals were involved, and no clinical trials were conducted. Images showing people together with the demonstrator are for illustrative purposes only. All devices containing electrical components were deactivated at the time.

After consultation with the Ethics Commission in Leipzig (Germany), and in accordance with section 15 of the professional code of conduct of the Saxony State Medical Association, no ethics approval was required for the work described.

### Proof-of-Concept Development

In the first phase of the development process, we identified requirements for a training system with digital assistance based on the wishes and challenges associated with home-based scoliosis therapy [13]. To address the lack of therapeutic support, low motivation, and limited space, the system should have the following features: a digital profile, exercise instructions, measurement functions to monitor exercise execution, low space requirements, modifiability for holistic training, and the integration of established but rarely used at-home training devices such as balance boards, wall bars, mirrors, and exercise sticks. We have transferred these requirements into a modular approach, which combines conventional devices with sensor functions, and further specified them (Table 1).

**Table 1.** Requirements specification for the 4 modules of the new training system.

Specification	Module 1	Module 2	Module 3	Module 4
Device	Mirror	Wall bar and exercise stick	Balance board	Mirror
Form	Smart textile and wearable	Modifiable training device	Balance training device	Sensor hub and web interface
Function	Monitoring exercise performance	Versatile training with minimal space requirements	Training proprioception, self-correction, and compensating for imbalances	Preparation and visualization of measurement data and output of exercise instructions
Tracking	Breathing (chest and abdomen) and movement (torso and head)	Pressure and gripping forces	Inclination and pressure distribution	None

For each of these modules, the names, designs, and sensor concepts were created in a second development phase. The first module was named “Wearable-Module” due to its design as a portable, functional textile that is primarily used for tracking. Modules 2 and 3 represent the training components, with the second module named “Bars-Module” because of its rod-shaped design and the third module named “Balance-Module” due to its resemblance to conventional balance boards. The fourth module is used to visualize all sensor data and acts as an interface between the user and the new system, which is why it was named “Interface-Module.” For the entire system, the acronym “iScoolio” was chosen, which combines the terms “scoliosis” and “school” and describes the process of “I school myself,” as the guiding principle of home-based scoliosis therapy.

In the final development phase, we implemented a demonstrator for each module, which is presented as the “iScoolio concept” in the *Results* section. A key component of this work was the development of a custom circuit board (iScoolio board). The board consists of a microcontroller (BlueNRG-355MT; STMicroelectronics N.V.), inertial sensors (BHI260AB and BMM150; Bosch), and a Wheatstone quarter bridge (*R*=10 kΩ), and it serves as a basis for all sensor functions. Powered by a lithium battery (LIR2032; AccuCell) and housed in an enclosure (70×34×17 mm), each circuit board can be used as a stand-alone unit. For wireless communication, each board has its own ID and radio channel and transmits its data every 6 milliseconds via its own 2.4 GHz frequency channel. This interval was chosen as a compromise between transmission speed and power consumption. The corresponding receiver scans each channel for 13 milliseconds to ensure data acquisition in case the first transmission fails. This resulted in a sampling frequency of 4 Hz.

### Theoretical Evaluation of Use Case Scenarios

A theoretical use case evaluation was conducted using the developed concept for the exemplary Schroth exercises “Between Two Poles,” “50X,” and “Sail” (Multimedia Appendix 2). Of particular interest were the variability, usability, and tracking capabilities of the system.

### Technical Verification

#### Overview

To test H_0_, a technical verification of the demonstrator’s measurement functions was conducted. This included motion tracking (H_1_) and respiration tracking (H_2_) of the Wearable-Module, force tracking in the handles and supporting feet of the Bars-Module (H_3_), and measurement of COP and detection of the stance width in the Balance-Module (H_4_). Test scenarios were developed for each of these functions, where the number of measurements was derived from the information for advanced users in the Schroth exercise manual (Multimedia Appendix 2). As identical hardware is used multiple times within a single measurement function, only a subset of the sensors used in the Wearable-Module and Bars-Module was tested on a sample basis. The data were measured using the iScoolio boards, with the Interface-Module serving as a wireless receiver. All measured values were recorded and stored in a CSV file using a data logging function of the Interface-Module. The data were analyzed and prepared graphically using the OriginPro software (version 2023; OriginLab Corporation).

#### Wearable-Module

##### Motion Tracking

To verify H_1_, a test bench was developed that can move to a specified position with repeatable accuracy. This consisted of a wooden table, 4 servo motors (2000 Series Dual Mode Servo [25‐2, Torque]; goBILDA) with 3D-printed pulleys and deflection rollers, a microcontroller (Uno R3; Arduino), and a distribution board (Servo Power Distribution Board [8 Channel]; goBILDA). Additional components were additively manufactured for the top of the table to serve as holders for 3 iScoolio boards. The boards could be arranged in a tower shape using a Velcro connection. The bottom board was fixed in place, while the top 2 were mounted on pressure springs for mobility. The concept thus resembled a greatly simplified spine with fixed elements (vertebrae/3D-printed components) and flexible elements (intervertebral discs/compression springs; Figure 1A). With the help of servo motors arranged at a 90° angle and wire ropes mounted at the top of the tower, the simplified spine structure could be moved 3D in space within the volume of a hemisphere.

**Figure 1.**
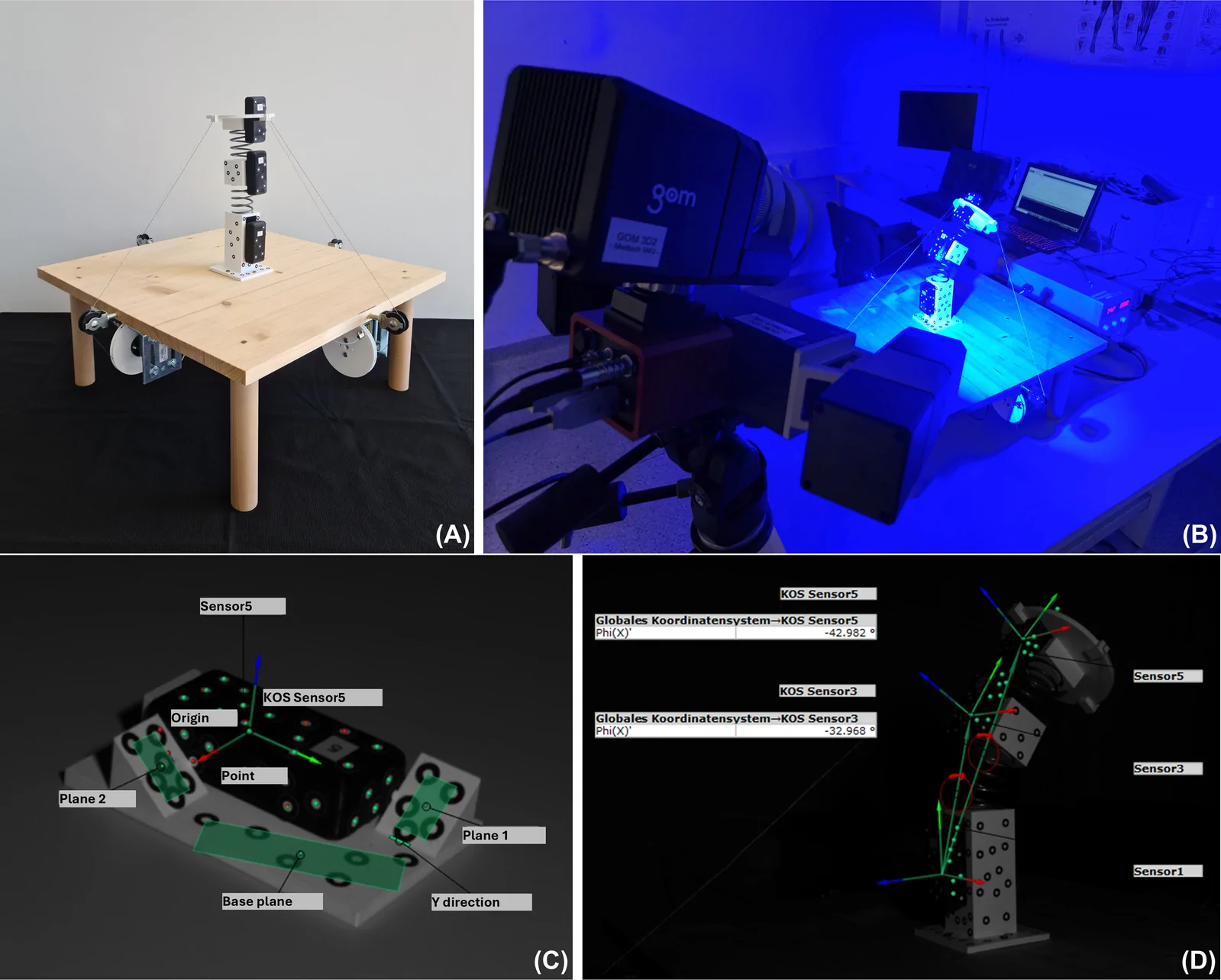
Verification of the Wearable-Module’s motion tracking. Actuator test bench designed as a simplified spinal model for reproducible motion of 3 iScoolio boards (A) in the neutral position and (B) during optical measurement with the ARAMIS 3D system. (C) Screenshot from the GOM Correlate Pro 2022 software illustrating virtual placement of a local coordinate system (KOS Sensor5) at the inertial measurement unit position on an iScoolio circuit board. (D) Screenshot showing the coordinate systems of sensors 3 and 5 during motion in the sagittal plane, including the calculated rotation angles relative to sensor 1.

Three iScoolio boards (1, 3, and 5) were attached to the test bench to verify motion tracking. These boards were selected to mirror the planned vertical arrangement and because sensor 1 serves as the reference for all the others. On the basis of the “50X” exercise, 2 positions in the frontal plane (yaw) and 2 positions in the sagittal plane (pitch) were then approached 50 times, with an intermediate return to the zero position, and held for 10 seconds each. In the frontal plane, the “spine” was tilted once to the left and once to the right, and in the sagittal plane, once anteriorly and once posteriorly. At the same time, the sensor data (Euler angles) were recorded.

A marker-based optical system (ARAMIS 3D camera, 600 mm measuring volume; Carl Zeiss GOM Metrology GmbH) served as a reference for this verification (Figure 1B). After covering all relevant surfaces with 3 mm measuring points, a manual image was taken at each position approached during the test run using a manual trigger.

As a basis for evaluating the recorded images, the actual sensor position of the inertial measurement unit (IMU) within the iScoolio board housing had to be determined by software. For this reason, an additional adapter was additively manufactured to enable the calculation (Figure 1C). For each of the 3 sensors, the graphically determined points were then defined as the origin of a local coordinate system in the 3D measurement software GOM Correlate Pro 2022 (Carl Zeiss GOM Metrology GmbH) associated with the ARAMIS 3D system.

Using these 3 coordinate systems, the inclination for the approached positions could be determined for sensors 3 and 5 in relation to sensor 1 (Figure 1D). In Figure 2, the angle measurements from both systems are depicted as box plots.

**Figure 2.**
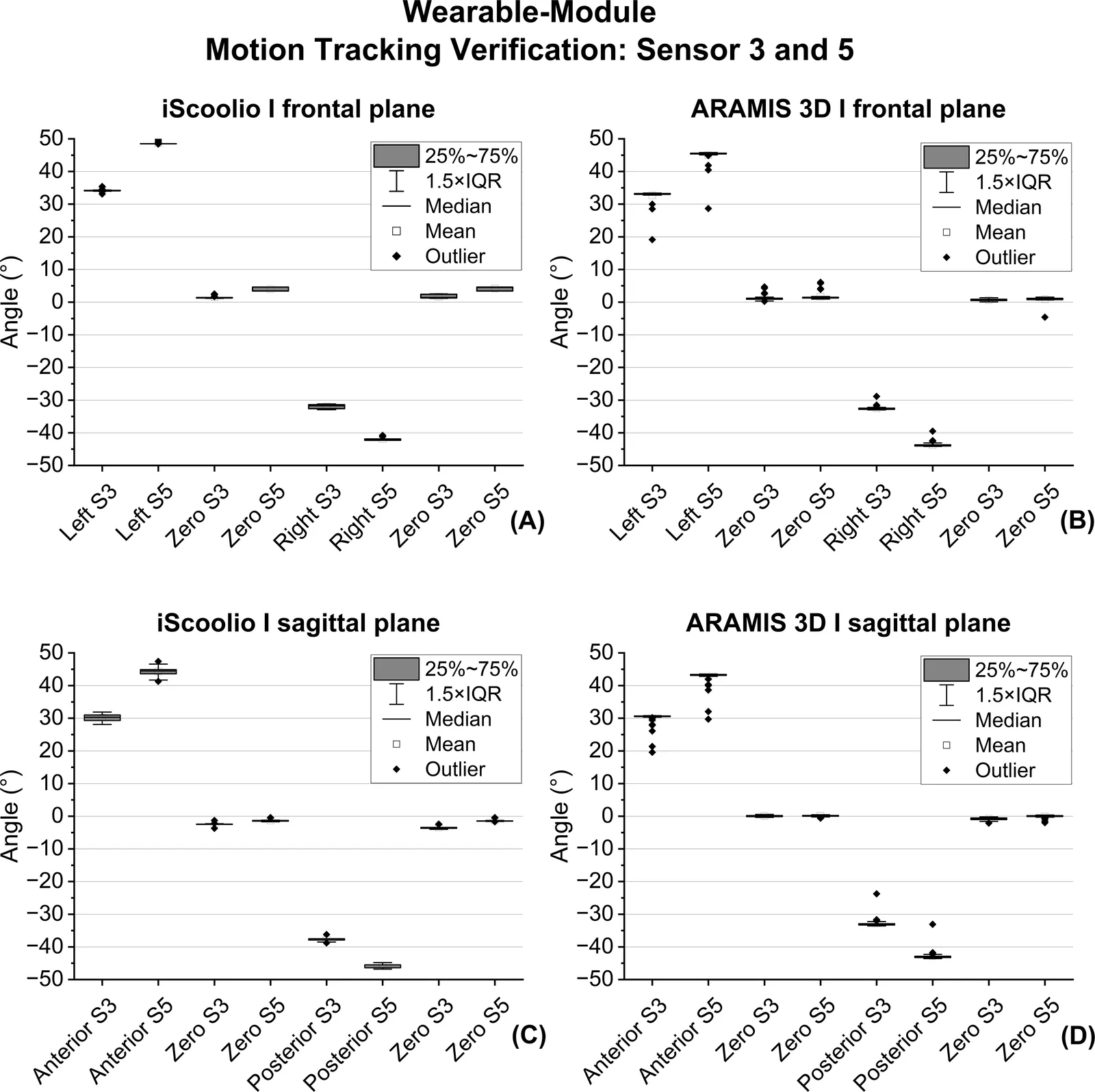
Verification of the Wearable-Module’s motion tracking. (A, B) Box plots of the measured angles of sensors 3 and 5 during repeated movement to 2 positions in the frontal plane, including return to the 0 position. (C, D) Box plots of the measured angles during repeated movement to 2 positions in the sagittal plane, including return to the 0 position. (A, C) Measured values of the Wearable-Module. (B, D) Measurements taken with the ARAMIS 3D system.

##### Respiration Tracking

A torso test bench with breathing simulation was set up for evaluating H_2_. To simulate breathing, 2 pneumatic mounting cushions were attached laterally to a mannequin, which generated a stroke of 75 mm at a pressure of 0.5 bar. This was intended to simulate the lifting of the chest during inspiration (Figure 3). The mounting cushions were filled and emptied using a proportional pressure control valve (VEAB-L-26-D07-04-A4-1R1; Festo), which was supplied with 3 bar of compressed air and controlled via an Arduino circuit.

**Figure 3.**
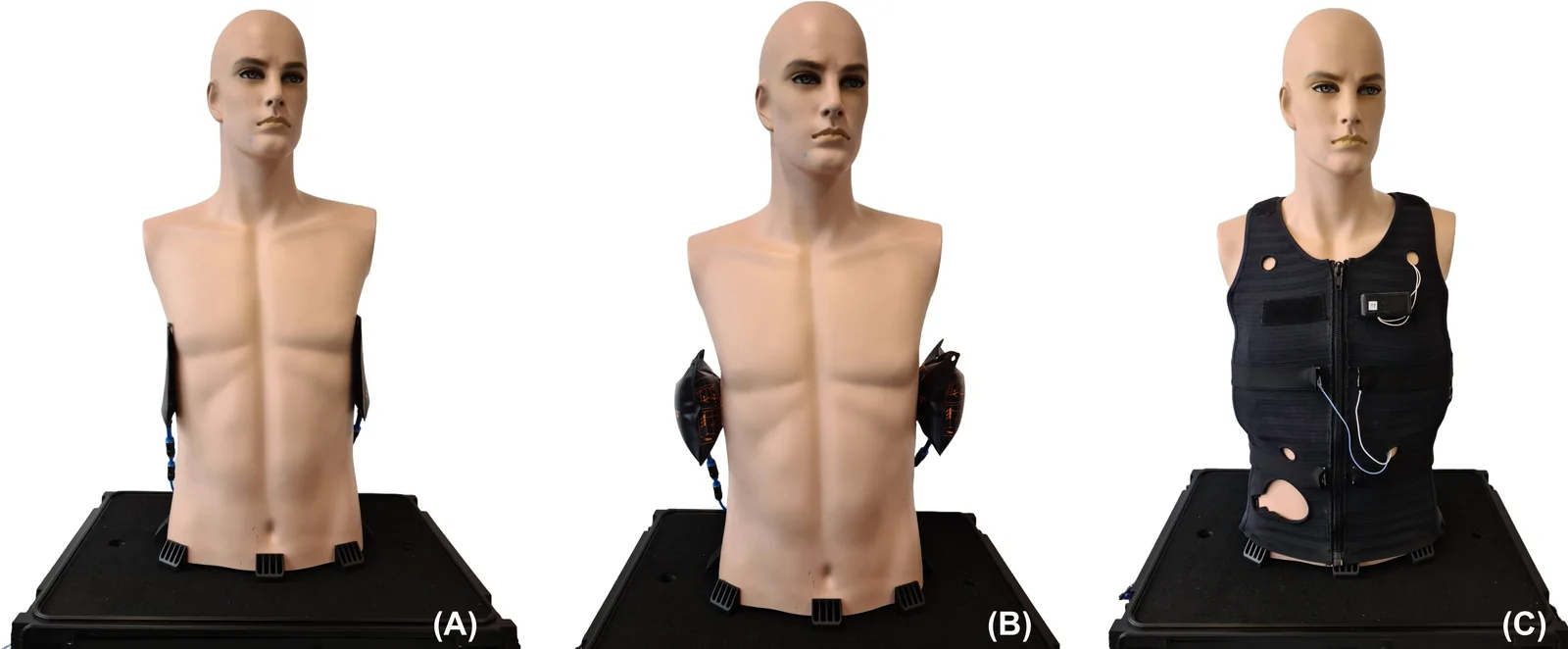
Verification of the Wearable-Module’s respiration tracking. A torso model with laterally applied air cushions served as the test device. (A) Expiration. (B) Inspiration without the Wearable-Module. (C) Inspiration with the Wearable-Module donned.

Using this test bench, 2 measurements with the Wearable-Module attached were carried out. The first test, “frequency measurement,” was designed to examine the detection of breathing cycles. On the basis of the “Sail” exercise, 40 breathing cycles (5×8 breaths) were simulated while recording the sensor data. In the second test, “amplitude measurement,” it was checked whether the depth of breathing could be determined. For this purpose, 40 cycles of shallow breathing (4 seconds of filling and 10 seconds of emptying) and deep breathing (6 seconds of filling and 15 seconds of emptying) were simulated while logging the measured values. The results of both experiments are shown in Figure 4. For the frequency measurement, the trigger curve was plotted in time synchronization with the measured values. For the amplitude measurement, 2 box plots were calculated. This calculation was based on the measured maxima for shallow and deep breathing in each of the 40 cycles. After checking the normal distribution of the differences between these 2 datasets, a 2-tailed paired *t* test (α=.05) was used to evaluate whether the difference was significant.

**Figure 4.**
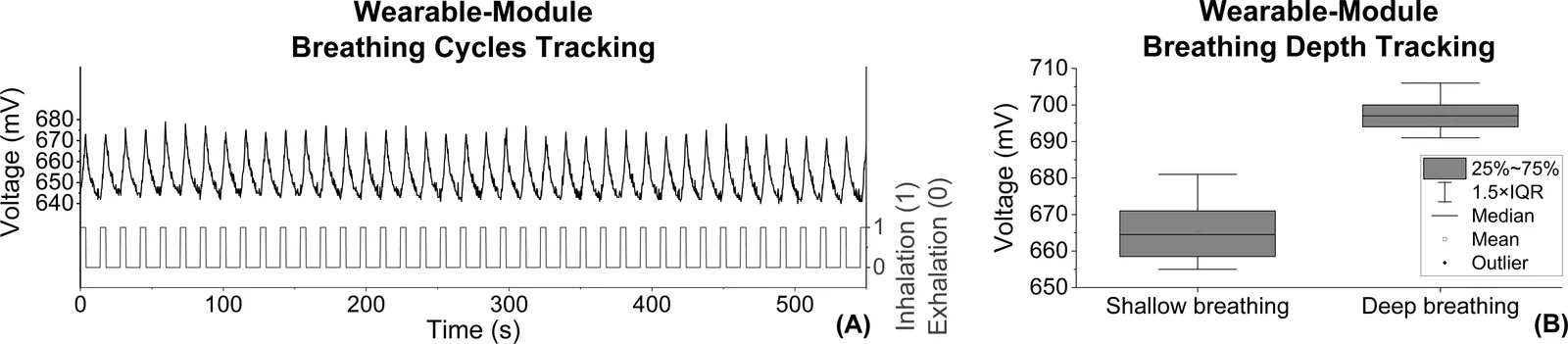
Verification of the Wearable-Module’s respiration tracking. (A) Measured values for 40 simulated breaths over time, including the trigger signal. (B) Measured values for simulated shallow and deep breathing in the form of 2 box plots.

### Bars-Module

#### Force Tracking: Handles

To verify H_3_, a test setup was designed for the handles of the Bars-Module that used a servo-electric testing machine (tensile/compression and torsion testing machine, 10 kN/200 Nm; DYNA-MESS Prüfsysteme GmbH) as a reference. The verification was conducted at 3 evenly spaced load levels to fully cover the sensor’s measuring range. The following test protocol was implemented for the testing machine for verification purposes:

1. Preconditioning: Apply 110% of the maximum force (484 N) to the sample, hold for 3 seconds, and then release. This procedure was repeated 5 times.
2. First force level: Apply one-third of the maximum force (147 N) to the test specimen and hold for 10 seconds, then release.
3. Second force level: Apply two-thirds of the maximum force (296 N) to the sample and hold for 10 seconds, then release.
4. Third force level: Apply the maximum force (444 N) to the test specimen and hold for 10 seconds, then release.
5. Repeat the cycle 40 times in total.

The cycle count of 40 is attributable to the performance of the “50X” exercise. To be able to mount the iScoolio handpiece on the testing machine, 2 half-shell adapters, designed to closely resemble a grasping hand, were additively manufactured. As a replacement for the rod-shaped base body of the Bars-Module, a round wooden profile (diameter 28 mm and length 19 cm) was also inserted into the handpiece to provide a counterbearing. After placing the handpiece in the testing machine (Figure 5), the test program was started, and the sensor data were recorded.

**Figure 5.**
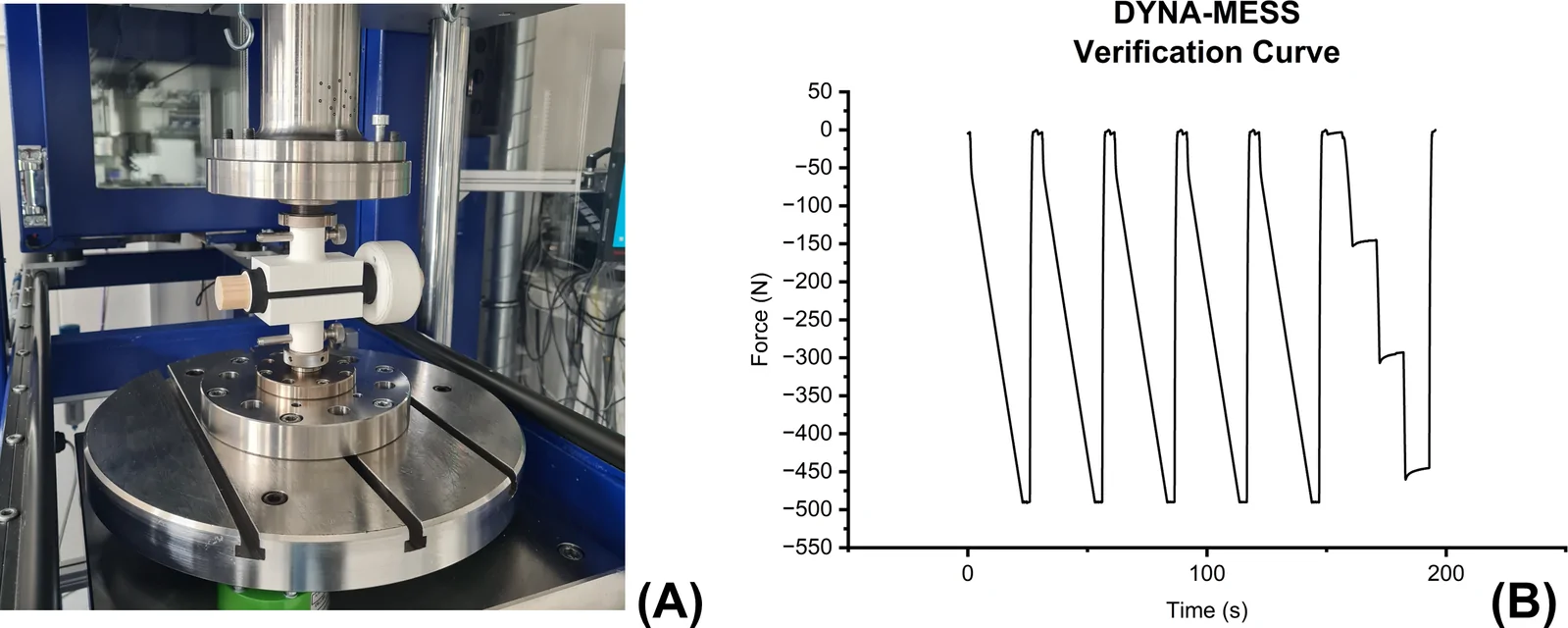
Verification of the Bars-Module’s force tracking (handles). (A) Measurement setup. (B) Verification curve of the testing machine.

To examine the differentiability of the force levels, repeatability, and drift, the measurement data were evaluated in the form of 2 diagrams. First, 3 box plots were created for the measured values of the 3 force levels. These were based on the calculated mean values of the 10-second holding phases, excluding the first and last seconds, to eliminate overswinging of the testing machine. Second, these calculated mean values were visualized in chronological order in a line diagram (Figure 6). To assess significant differences among the 3 force levels in a within-subject design (N=40, k=3), we used the Friedman test. The Dunn test was subsequently applied for post hoc pairwise comparisons following a significant Friedman result. Significance was set at an α of .05. The choice of this procedure reflects the within-subject structure (3 related samples) and the nonnormality of the differences among the 3 conditions, as indicated by the Shapiro-Wilk test (α=.05).

**Figure 6.**
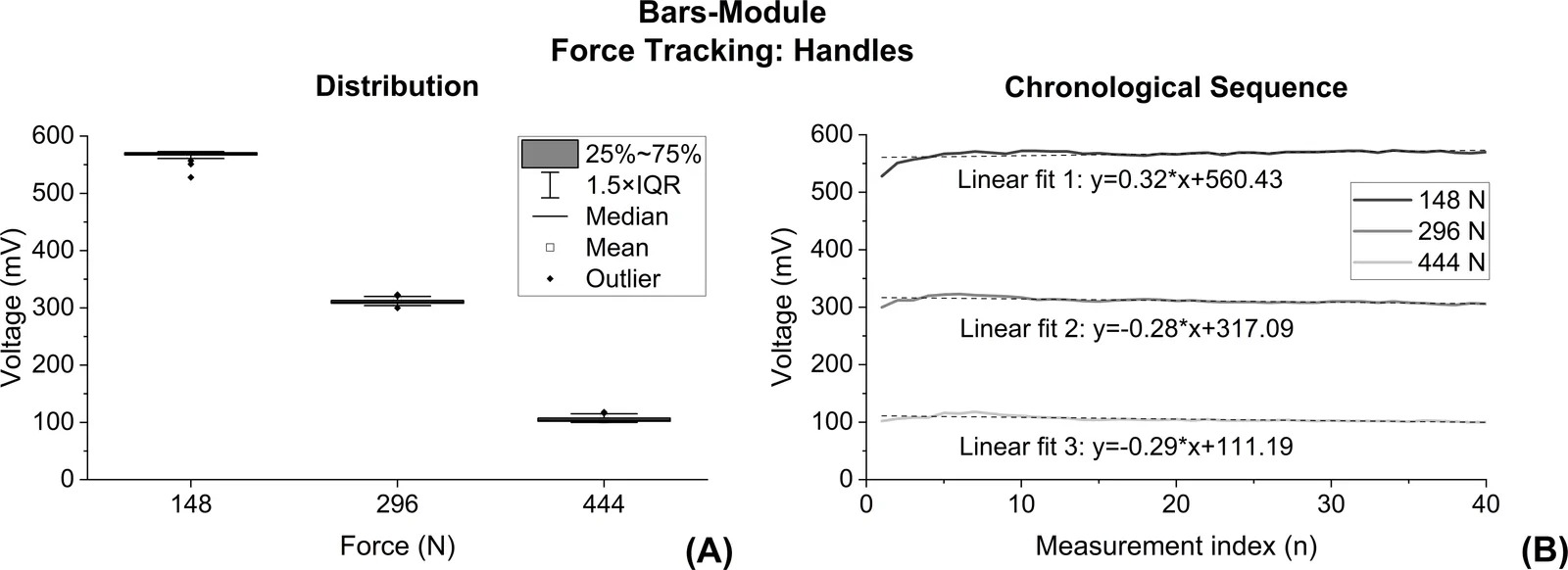
Verification of the Bars-Module’s force tracking (handles). Measured values for the 3 force levels are visualized in the form of (A) 3 box plots and (B) a line diagram.

#### Force Tracking: Supporting Feet

Similar to the measurements of the iScoolio handles, the supporting feet were also verified using the servo-electric testing machine, contributing to the investigation of H_3_ (Figure 7). Due to the slightly larger force range of the sensors installed in the bases, the test protocol for the handpieces was adjusted to a maximum force of 445 N. In addition, the number of cycles was reduced to 25, corresponding to the repetitions (5×5) for the “Between Two Poles” exercise. The analyzed measurement data are shown in Figure 8.

A Friedman test for related samples (N=25, k=3) assessed differences across the 3 force levels, and the Dunn test was subsequently applied for post hoc pairwise comparisons, with an α of .05. The test procedure was selected in the same way as for the handles.

**Figure 7.**
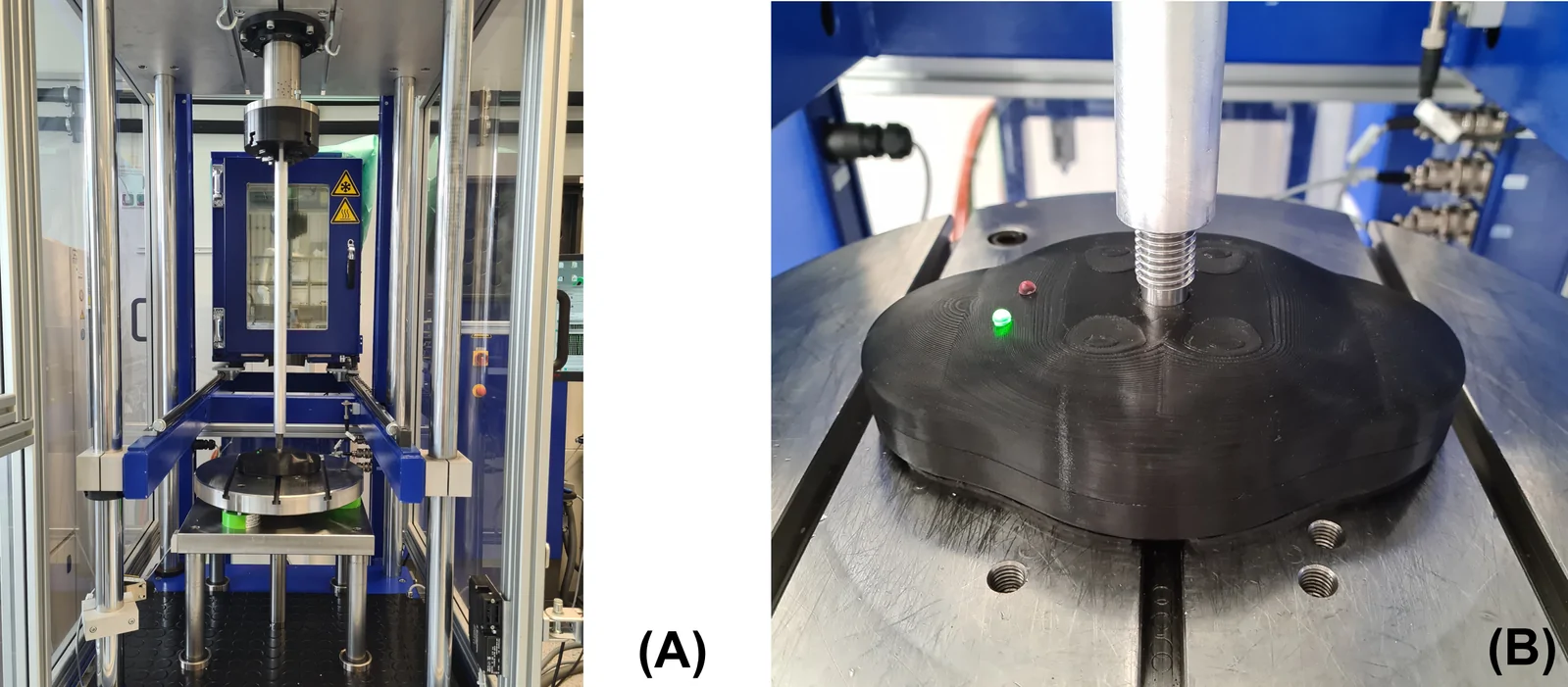
Verification of the Bars-Module’s force tracking (supporting feet). The test setup is shown in 2 different views (A and B).

**Figure 8.**
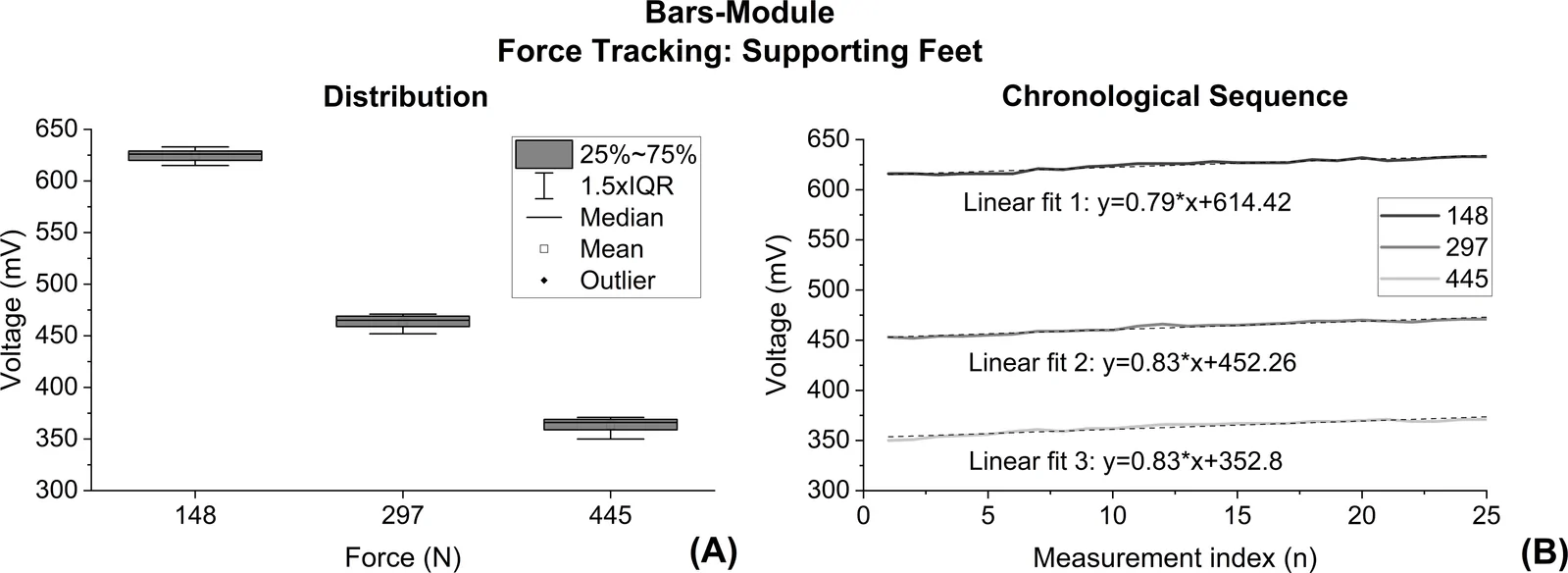
Verification of the Bars-Module’s force tracking (supporting feet). The measured values for the 3 force levels are visualized in the form of (A) 3 box plots and (B) a line diagram.

### Balance-Module

#### Stance Width

The evaluation of H_4_ involved examining 2 measurement functions of the Balance-Module. To assess the first function, stance width measurement, the board was locked in the neutral position using the base feet. In addition, the measurement surface was covered with a transparent film so that reversible markings could be made. For the verification, 2 test weights (5 kg) were placed on the board so that they touched each other and were then manually pushed apart until the Interface-Module indicated a color change for the minimum distance being reached (Figure 9). The positions of the weights were then marked on the transparent film in color, and their shortest distance was measured. This test procedure was performed 50 times. The corresponding box plot is shown in Figure 10.

**Figure 9.**
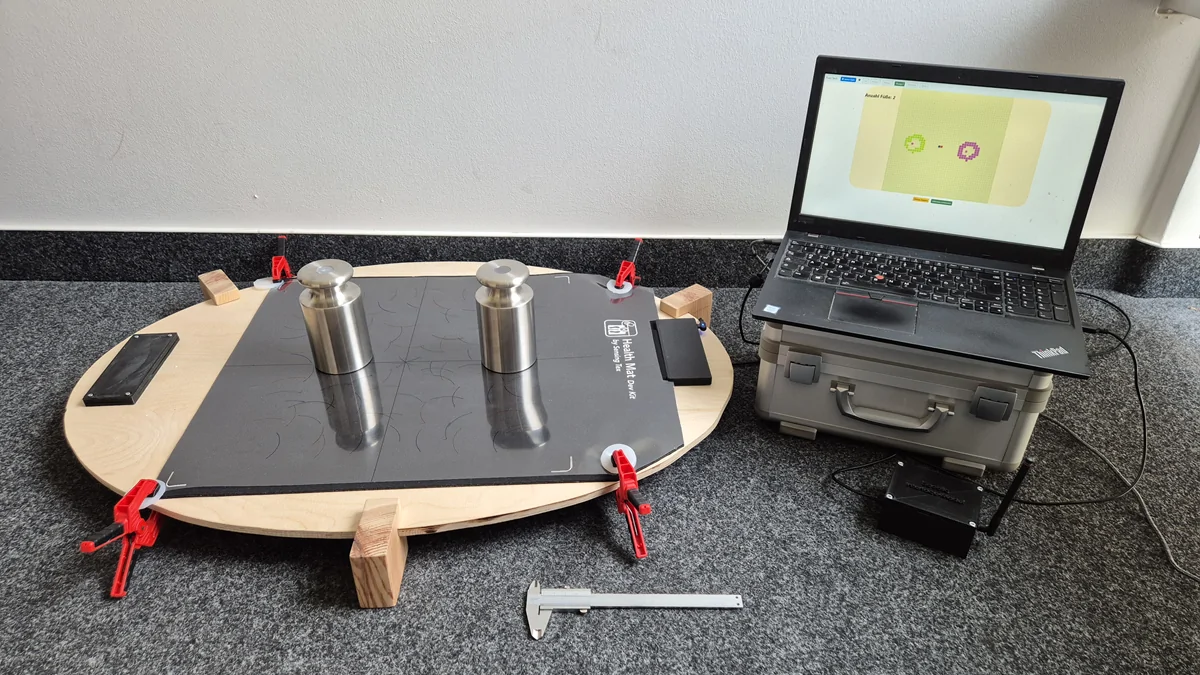
Verification of the Balance-Module’s stance width tracking. The test setup consists of the locked Balance-Module with applied film and 2 test weights, as well as the Interface-Module.

**Figure 10.**
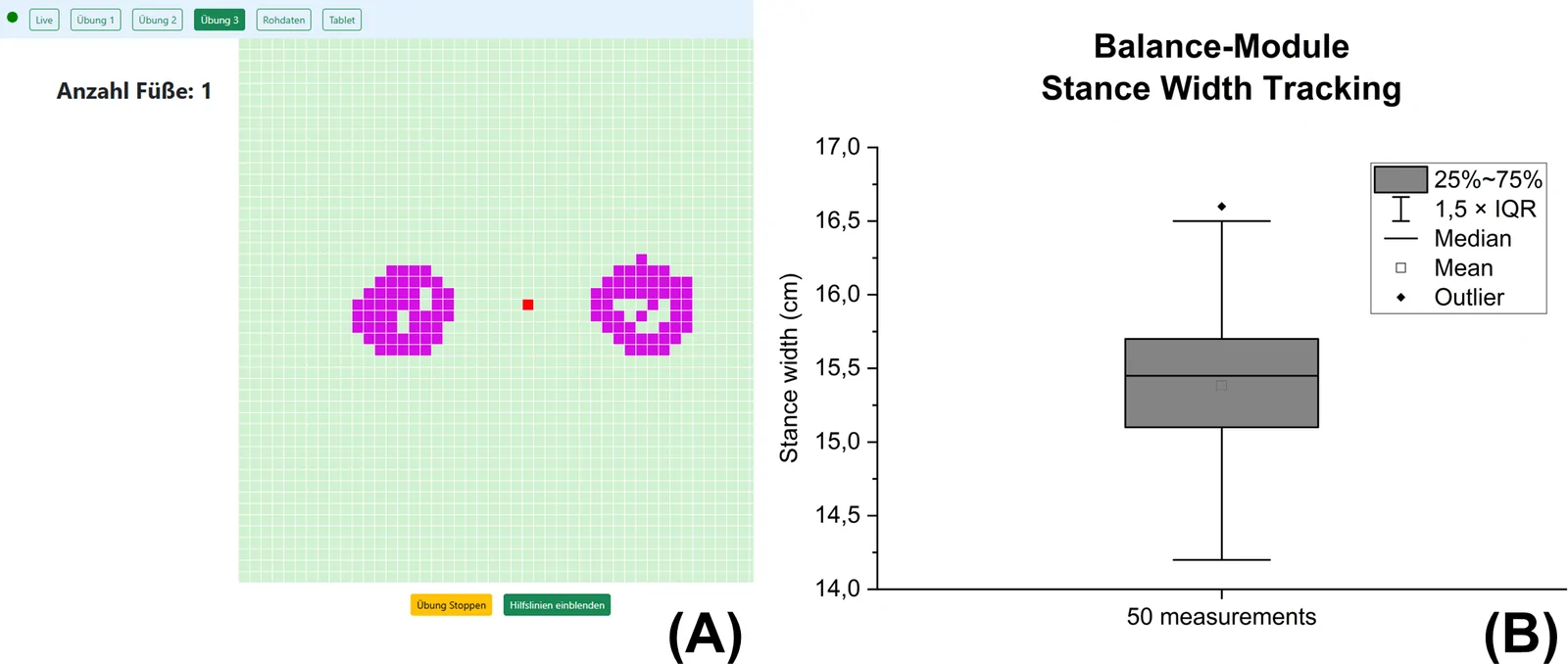
Verification of the Balance-Module’s stance width tracking. (A) Screenshot of the Interface-Module with test weights that are too close together, as indicated by the same color appearing twice. (B) Box plot for measuring stance width over 50 passes.

#### Center of Pressure

To verify the second measurement function, the COP measurement, the board was also locked in a neutral position, and the transparent film was marked with horizontal and vertical lines spaced 4 cm apart. This distance was determined by the diameter of the 5-kg test weights. While maintaining the minimum distance, 2 of these test weights were placed at 50 different positions along the grid on the board, always in a horizontal alignment to simulate a normal 2-foot stance. A screenshot was then taken, and the boxes between the local COPs and the global COP were counted (Figure 11A). These were subtracted from each other and halved to determine the deviation from the target value in terms of boxes. Due to the equal weight of the 2 test weights, the global COP should be exactly in the middle between the local COPs. The histogram for the 50 measurements is shown in Figure 11B.

**Figure 11.**
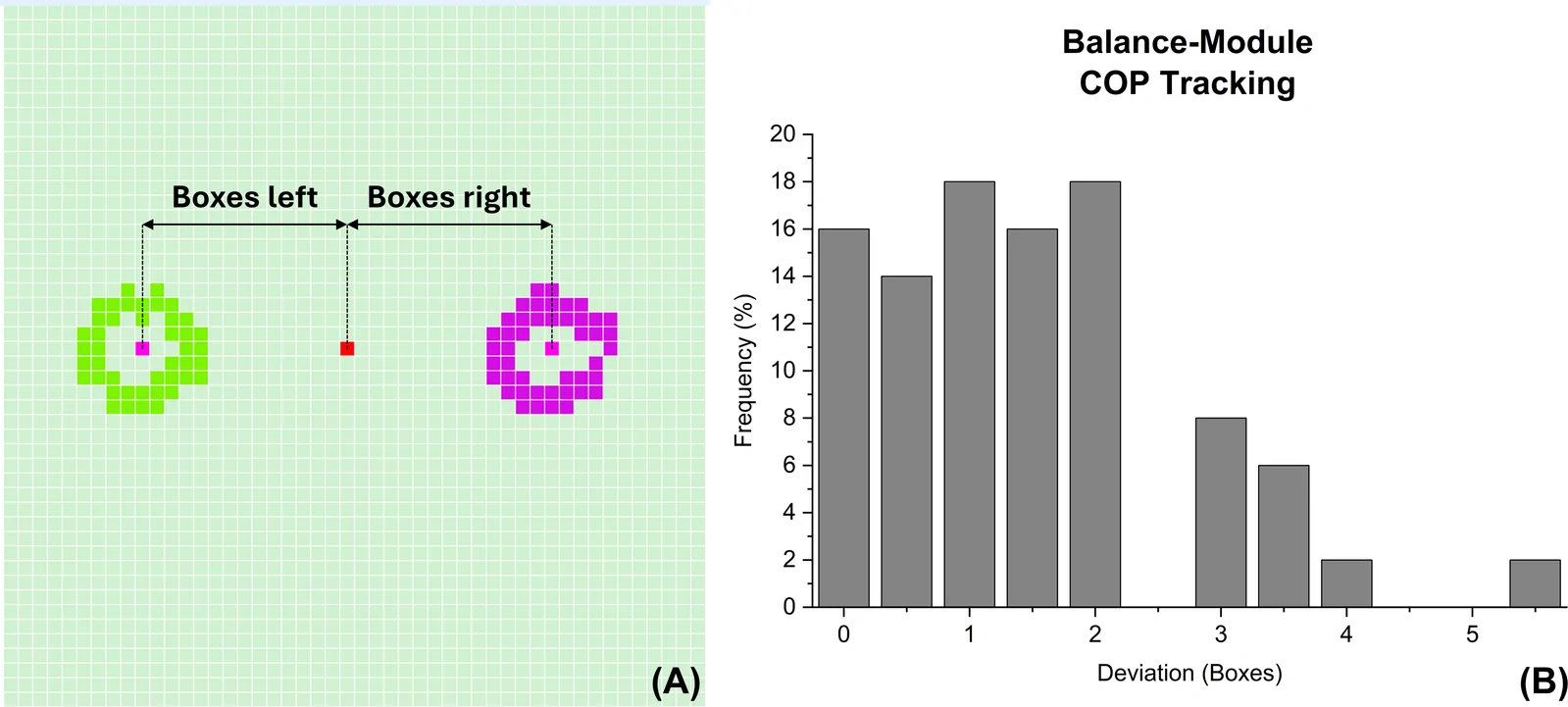
Verification of Balance-Module’s center of pressure (COP) tracking. The figure shows a screenshot of the Interface-Module (A) from 1 of the 50 measurements with 2 local COPs of the clusters and a global COP of the entire measurement area (red dot). The double arrows show the distances between the COPs. The histogram (B) visualizes the frequency of the deviations determined in boxes of the global COP (direction unspecific) from the target value. The value 0 thus describes the perfectly centered alignment of the global COP between the 2 local COPs.

## Results

### iScoolio Concept

#### Wearable-Module

The Wearable-Module consists of a headband and a vest with elastic elements on the sides to adapt to the wearer’s body shape (Figures 12B and 12C). Two resistive textile strain sensors, each connected to an iScoolio board, record the circumferential changes of the chest and abdomen during breathing. This allows both inspiration and expiration, as well as the relative depth of breathing, to be detected. For motion tracking, the module contains a total of 9 iScoolio boards, 1 on the headband and 8 distributed across the back. The positioning on the back was based on the 4-block model used in Schroth therapy. For each block, a total of 2 iScoolio boards were placed parallel at the same height on the back (Figure 12A). The movement data are derived based on quaternions. iScoolio board 1, which was placed on the left side of the hip-pelvic block, serves as a reference for all other motion tracking sensors, as there is less movement in this position compared to the other positions further up. The Wearable-Module thus serves as a tracking component for movement and breathing.

**Figure 12.**
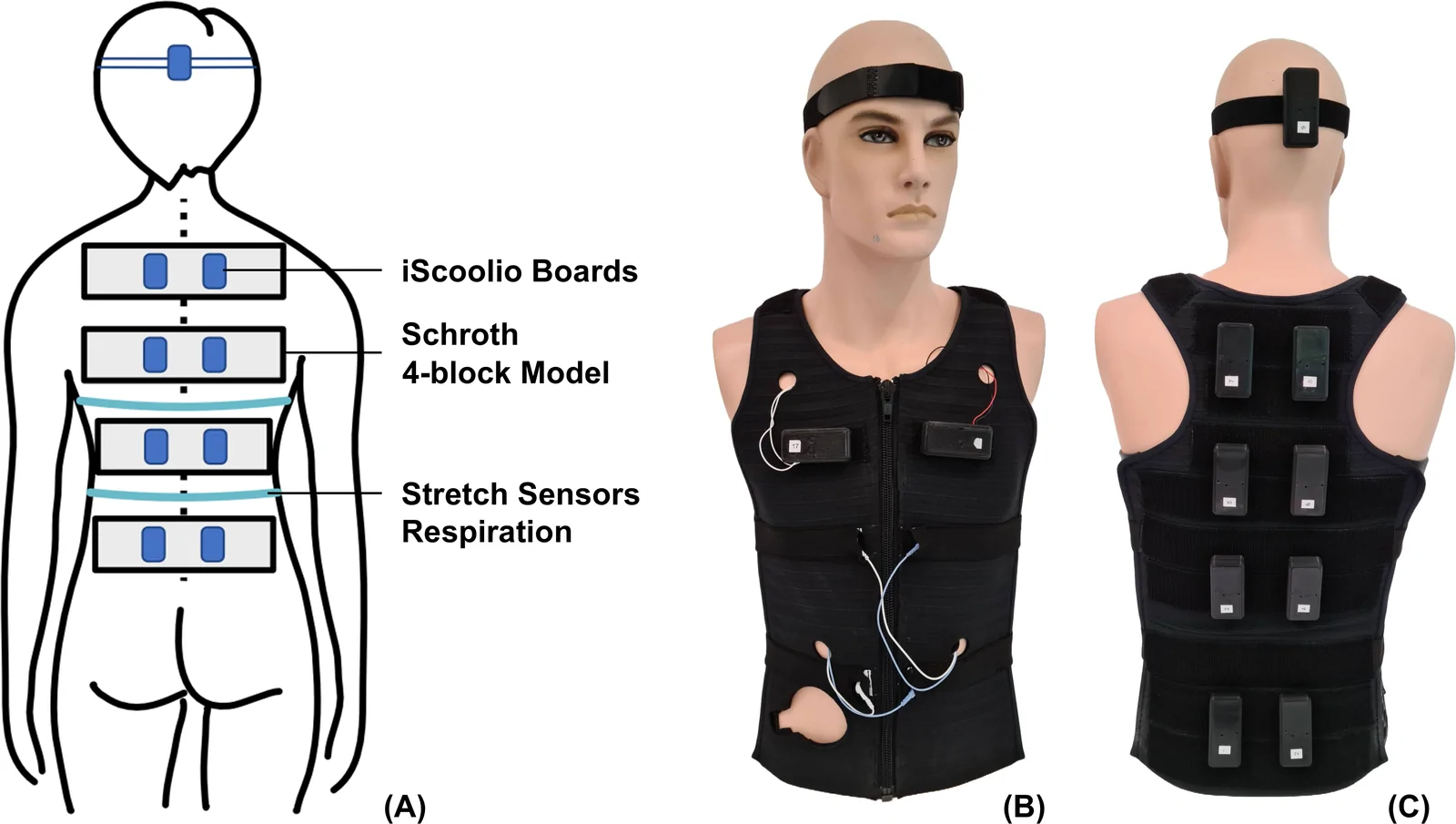
(A) Schematic placement of the iScoolio boards and strain sensors in the Wearable-Module. (B, C) Transfer to a functional prototype based on the 4-block model according to Lehnert-Schroth [1].

#### Bars-Module

The Bars-Module consists of 2 bars with attachments and combines the functions of exercise sticks and wall bars. Both bars are made of an aluminum profile and have an internal thread on both ends. Using a left-hand and a right-hand thread and the corresponding threaded rods, the Bars-Module can be braced in the door frame to simulate a wall bar (Figures 13A and 13B). It can also be used as an exercise stick by changing its length with an extension piece (Figure 13C). To simulate the wall bars, supporting feet with an antislip coating have been designed, which provide additional support when clamped in the door frame (Figure 13D). The Bars-Module is completed by sliding handles, which are also equipped with a special antislip coating to ensure a secure grip. Resistive force sensors were integrated into both the handles (FlexiForce A502; Tekscan) and the supporting feet (FlexiForce A201; Tekscan). These enable touch-specific (eg, “reach for the rungs”) as well as force- and intensity-specific training instructions (eg, “push both bars into the floor as you exhale”) to be followed. In addition, the clamping force of the Bars-Module can be measured when it is clamped into the door frame. The sensors are read, and the data are transmitted wirelessly via an iScoolio board, which has been integrated into each attachment. As an additional safety feature, the Bars-Module also offers the option of providing visual feedback on the clamping force via an LED integrated into the supporting feet (Figure 13D). It thus functions as a configurable and versatile training device for the new therapy system.

**Figure 13.**
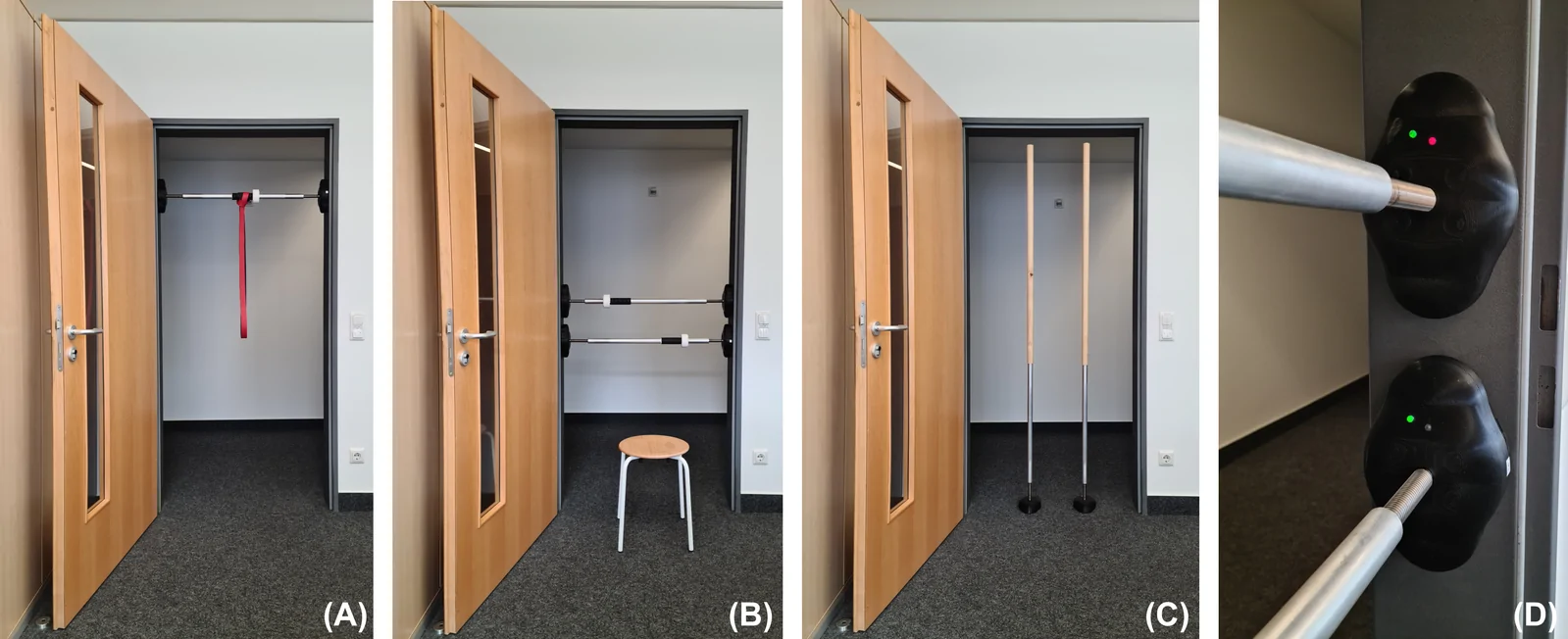
Use of the Bars-Module as a (A) single rung with a gymnastic band, (B) wall bar replacement with 2 rungs, and (C) long exercise sticks. (D) The green and red LEDs inside the supporting feet can be used to provide visual feedback to the user.

#### Balance-Module

The Balance-Module was based on the design of existing balance boards. To achieve the desired unstable surface, an oval-shaped base plate was made of wood and fitted with a 2-part, 3D-printed plastic element at the center of the underside. This consisted of an adapter plate with an iScoolio board mount and a screw-on ball segment (Figures 14A and 14B).

**Figure 14.**
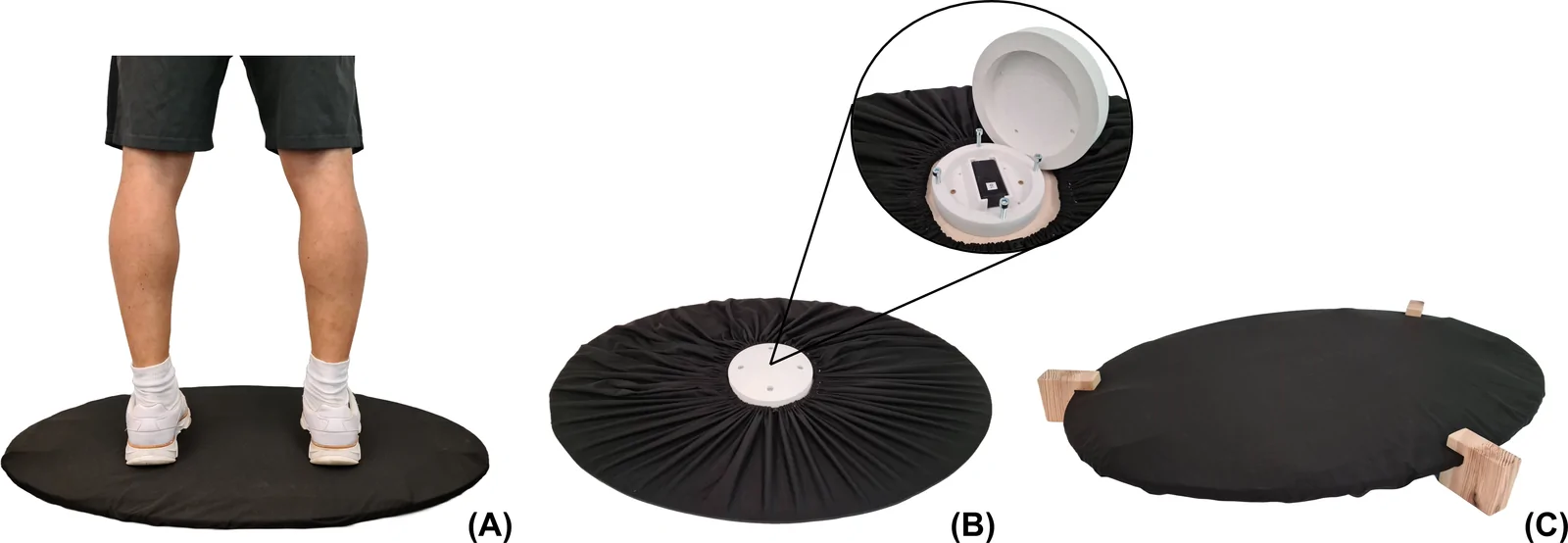
(A) Top and (B) bottom of the Balance-Module with (C) feet for targeted decoupling of mobility. The iScoolio board is integrated via the additively manufactured ball segment, which was designed to be modular and with an associated socket (B, detailed view).

The typical balance training device was expanded to include sensor components, which are of central importance for guiding and monitoring exercises in our development. On the basis of the structural and functional requirements of a balance board, 2 monitoring parameters were selected: the inclination of the board and the pressure distribution on the standing surface. The former was achieved by integrating the iScoolio board into the adapter plate. For the latter, a pressure measurement mat with 2304 sensor elements (Health Mat Dev Kit 1.9; Sensing Tex) was applied to the base plate. Specific algorithms were implemented to measure the number of feet on the board, the foot pressure distribution, the stance width, and the COP. When the sensor surface is loaded, adjacent sensor elements are combined into a cluster by the software while maintaining a specified maximum distance. A local COP is then calculated for each of these clusters, and the pressure distribution is visualized. On the basis of the intended application scenario, a detected cluster is recognized as a single foot. When a second cluster is detected, an additional algorithm checks the distance between the two. If this exceeds a specified value, the output indicates that a second foot is on the board. With the help of this parameter, the minimum stance width can be determined and tracked. Finally, an algorithm was implemented that calculates and visualizes a global COP for the entire sensor surface. To prevent the board from moving during these measurements, wooden feet were developed that can be attached to lock the Balance-Module in a horizontal position (Figure 14C). Conversely, it is also possible to shift the COP in a targeted manner as part of a PSSE exercise. To protect it from external influences and enhance its appearance, the Balance-Module was finally encased in a cover made of black functional jersey (PA 6.6). Alongside the Bars-Module, it is the second training device of the new system.

#### Interface-Module

In terms of hardware, the Interface-Module consists of 2 physically separate systems: a data processing unit (DPU; MeLE Mini PC Quieter 3Q Celeron N5105; Shenzhen MeLE Digital Technology Ltd) with a separate Bluetooth receiver (BlueNRG-LP; STMicroelectronics N.V.) and an output device, such as a standard mobile phone or tablet as a portable display device. Communication between the 2 components takes place via a wireless local area network data connection, while communication with the other modules takes place via Bluetooth. The software developed for the Interface-Module consists architecturally of several logical individual components, with data processing carried out in Python (Python Software Foundation) and the implementation of the user interface based on TypeScript (Microsoft Corp) and the Angular (Google) framework. A dashboard was set up to visualize all sensor data (Figure 15).

**Figure 15.**
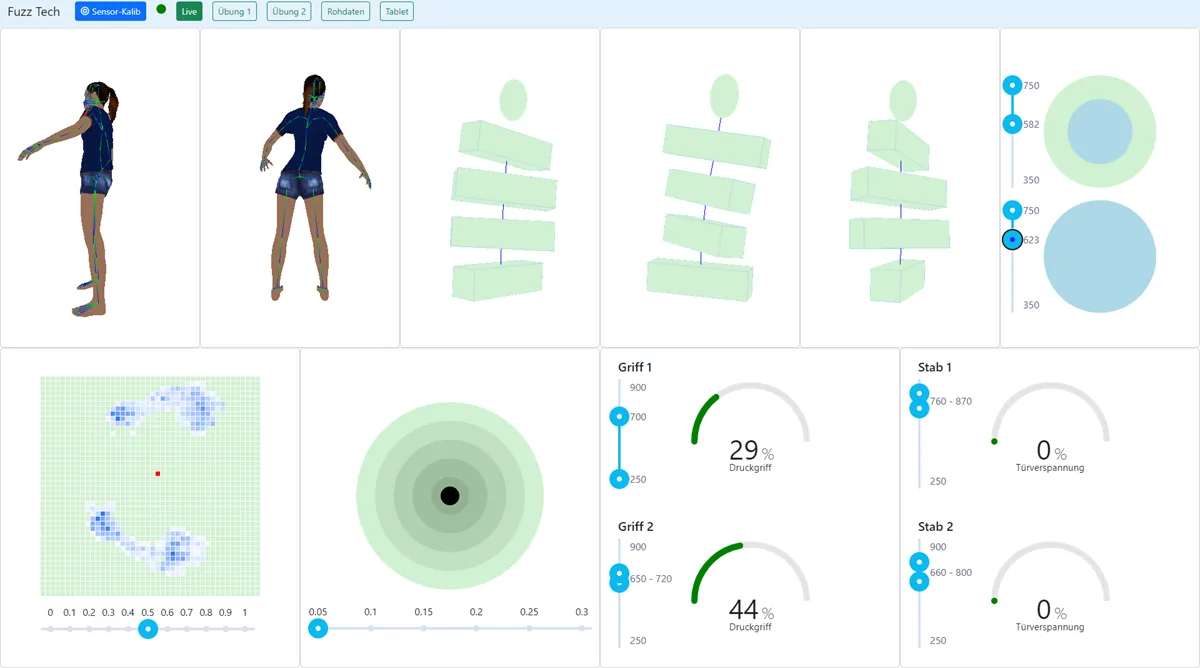
Dashboard of the iScoolio Interface-Module.

To implement therapy, the user selects an exercise from the catalog and starts it. Exercise performance is monitored by the DPU, and sensor data received via Bluetooth are evaluated against target parameters. The user receives real-time visual feedback on body position and exercise-specific instructions. A basic safety monitor protects against harm from system or application errors. If a sensor malfunctions, data transmission is interrupted, or tolerances are exceeded, the system enters an error mode with audible and visual warnings.

#### Theoretical Evaluation of Use Case Scenarios

In a thought experiment, the potential applications and limitations of the iScoolio concept were examined using the 3 exemplary Schroth exercises, “Between Two Poles,” “50X,” and “Sail.” As a result, Table 2 lists which modules can be used for the respective exercises and what functions they fulfill. Figure 16 shows what the use of the system might look like during the performance of the exercises.

With the current state of development, the following limitations would apply to the intended application scenario: a lack of motion tracking for the arms and legs; a low sampling rate of 4 Hz; a lengthy setup process due to the modular design and the fact that all iScoolio boards must be activated and calibrated separately; and unsuitability for exercises performed in the supine position due to the sensor housings on the back.

**Table 2.** Possible use cases of the iScoolio modules for 3 exemplary Schroth exercises.

iScoolio modules	Between Two Poles	50X	Sail
Wearable-Module	Tracking: upright posture and breathing	Tracking: alignment of the back and breathing	Tracking: upper body and head position and breathing
Bars-Module	Function: support elements; tracking: support force	Function: wall bars, tablet holder; tracking: grip strength	Function: support element; and tracking: support force
Balance-Module	Function: unstable surface; tracking: balancing and number or position of feet	Optional function: wobbly seat pad; tracking: balancing	Function: unstable surface; and tracking: balancing and number or position of feet
Interface-Module	Providing exercise instructions, visualizing measured values, and providing feedback on exercise performance	Providing exercise instructions, visualizing measured values, and providing feedback on exercise performance	Providing exercise instructions, visualizing measured values, and providing feedback on exercise performance

**Figure 16.**
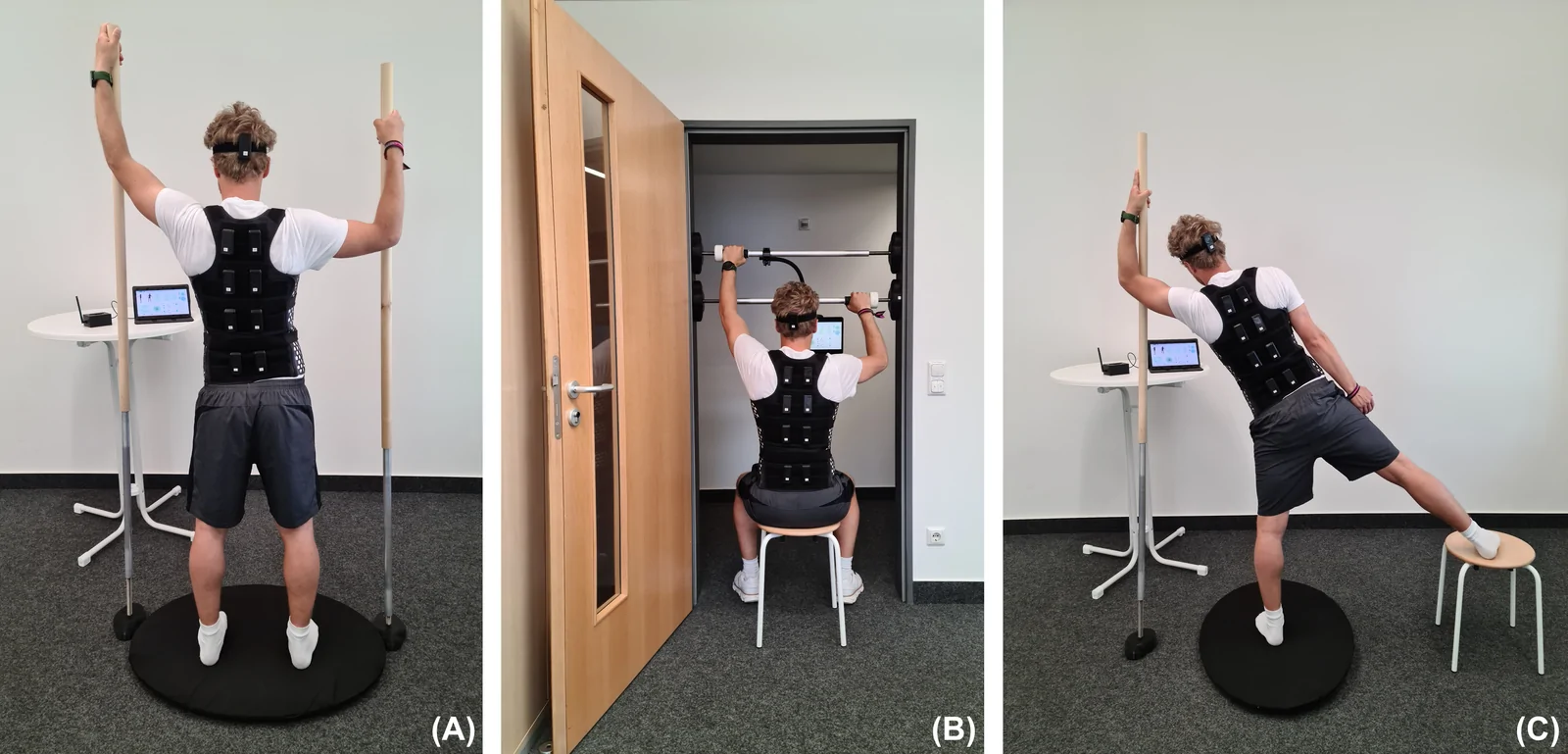
Potential applications of the iScoolio modules for the following sample exercises: (A) “Between Two Poles,” (B) “50X,” and (C) “Sail.”

### Verification of the Measurement Functions in the Laboratory

#### Wearable-Module

##### Motion Tracking

For all 4 positions approached, the mean values showed an absolute deviation between iScoolio and ARAMIS 3D of 2.29° (SD 1.66°). The largest deviations were observed in the posterior movement on the sagittal plane. The mean values of the zero positions were positive for both measuring systems in the frontal plane (iScoolio: 2.28°, SD 1.44° and ARAMIS 3D: 1.09°, SD 0.42°) and negative in the sagittal plane (iScoolio: −2.11°, SD 1.03° and ARAMIS 3D: −0.16°, SD 0.43°), with larger deviations occurring in the iScoolio demonstrator. The maximum SD in the iScoolio system was 1.48° (sagittal, S5, anterior; Multimedia Appendix 3). The measured values in the frontal plane were more accurate than those in the sagittal plane. The ARAMIS 3D reference system produced more pronounced outliers, which always affected sensor 3 and sensor 5 equally in the corresponding measurements (Figure 2).

##### Respiration Tracking

From the examination of the breathing cycle plot, the 40 simulated cycles can be clearly extracted from the sensor data provided by the Wearable-Module. For each cycle, there is a jagged curve with a rising flank during inhalation, a falling flank during exhalation, and a maximum at the change between the 2 states in this order. Fluctuations and outliers occur in particular when the filling volume of the artificial lung is low and during the transition between exhalation and inhalation.

For breathing depth, 2 datasets were obtained from the measured maxima: the mean for shallow breathing was 665.0 (SD 7.4) mV, and the mean for deep breathing was 697.4 (SD 3.7) mV. A paired *t* test of 40 paired measurements yielded a *t* value of −27.4 (df=39; *P*<.001), indicating a 2-tailed significant difference between the maxima. Despite all this, there is a high degree of variation within the 2 groups, which decreases with increasing breathing depth and thus increasing stretching of the sensor (Figure 4).

### Bars-Module

#### Force Tracking: Handles

The diagrams show 3 clearly separated measurement sets, corresponding to the 3 force levels: mean 567.1 (SD 7.5) mV at 148 N; mean 331.4 (SD 5.0) mV at 296 N; and mean 105.2 (SD 4.6) mV at 444 N. A Friedman test indicated significant differences among these levels: *χ*²_2_=80.0; *P*<.001. Dunn post hoc tests revealed significant pairwise differences: level 1 vs level 2 (*Z=*4.47; *P*<.001), level 1 vs level 3 (Z=8.94; *P*<.001), and level 2 vs level 3 (*Z*=4.47; *P*<.001). Within the box plots, only a few outliers are present. Furthermore, for the 3 equally spaced force levels (ΔF constant), the measured sensor data are not proportional, suggesting a nonlinear sensor response. Moreover, concerning the line diagrams, after a settling phase, a slight drift is observed during the approximately 2-hour and 13-minute test procedure, with a load-dependent drift direction, as indicated by the following slopes *m* and correlation coefficients *r*: *m*=0.32 and *r*=0.50 at 148 N; *m*=−0.28 and *r*=−0.64 at 296 N; and *m*=−0.29 and *r*=−0.74 at 444 N (Figure 6).

#### Force Tracking: Supporting Feet

The measurements exhibited a pattern similar to the handles. Across the 3 reference forces, no linearity was present in the 148 to 445 N range. The corresponding values for the 3 force levels are as follows: mean 624.7 (SD 6.0) mV at 148 N; mean 463.0 (SD 6.3) mV at 297 N; and mean 363.6 (SD 6.4) mV at 445 N. A Friedman test indicated significant differences among these levels: *χ*²_2_=50.0; *P*<.001. Dunn post hoc tests revealed significant pairwise differences: level 1 vs level 2 (*Z*=3.54; *P*<.01), level 1 vs level 3 (*Z=7.07*; *P*<.001), and level 2 vs level 3 (*Z=*3.54; *P*<.01). The box plots show no outliers with an IQR of 1.5. Compared to the handles, higher measuring ranges can also be observed for the 3 forces. The time series shows a roughly linear drift during the approximately 1-hour and 23-minute test procedure, with a positive drift direction, as indicated by the following slopes *m* and correlation coefficients *r*: *m=*0.79 and *r*=0.96 at 148 N; *m*=0.83 and *r*=0.97 at 297 N; and *m*=0.83 and *r*=0.95 at 445 N (Figure 8).

### Balance-Module

#### Stance Width

When examining the stance width, the 50 measurements resulted in an average value of 15.4 (SD 0.54) cm. The box plot also shows a narrow IQR (<1 cm) and only 1 outlier at 1.5× IQR. The range of the measurement is 2.4 cm (Figure 10).

#### Center of Pressure

During technical verification of the Balance-Module’s COP measurement function, the 50 measurements revealed an average deviation of 1.5 (SD 1.2) boxes. The histogram shows that the deviation was ≤1 box in 48% of the measurements and ≤2 boxes in 82% of the measurements. With a hardware center distance of 10 mm between 2 sensor elements or boxes, 82% of the measurements had a COP deviation of ≤20 mm. The largest deviation was 5.5 boxes. Furthermore, it has been shown that the edge areas of the sensor surfaces in particular lead to measurement errors when determining the COP. If the statistical measures were calculated for all measurements in which at least one test weight touched the edge of the sensor surface (n=18), the result was a mean value of 2.6 (SD 1.3) boxes. For all other measured values (n=32), the mean value was 0.9 (SD 0.7) boxes (Figure 11).

## Discussion

### Principal Findings

This paper introduces a concept for a home-based scoliosis training system, presented as a demonstrator and verified technically. The concept builds on findings from a previous online survey on scoliosis therapy, with a particular focus on independent training at home. Consisting of 4 modules, it enables varied training in a space-saving design, including sensor-based monitoring of exercise execution and the output of instructions without the need for additional conventional training devices. Through dedicated test scenarios and test benches, we verified the implemented measurement functions. The results confirm that the sensor framework yields valid and accurate measurements compared to technical references, in line with H_0_. The development can therefore be classified at Technological Readiness Level 4. A comparison with other approaches to scoliosis therapy found in the literature is presented in Table 3. The following sections discuss the selected subcomponents of the iScoolio concept.

**Table 3.** Comparison of the iScoolio concept with other systems described in the literature.

Study ID	Motion tracking	Respiration tracking	Force tracking upper limb	BPD^a^	App or web interface	Training concept
“iScoolio”	9×IMU^b^	2×stretch sensor	4×force sensor	Pressure mat	ME^c^, EG^d^, DS^e^	FT/IE^f^
“Wearable Scoliosis Monitoring System” [7]	3×IMU	—^g^	—	—	—	FT/EE^h^
“Scoliosis Rehabilitation Assistant” [5]	3×IMU	—	—	—	ME, EG, GF^i^, DS	FT/EE
“SpineCurer” [6]	3×IMU	—	—	—	ME, EG, GF, DS	FT/EE
“Wearable Sensor Array” [4]	3×stretch sensor	—	—	—	ME, EG, DS	FT/EE
“Disc4Spine” [8,9]	1×time-of-flight camera, 1×Empatica E4 (Empatica Inc), pelvis belt (N/A)	—	—	Force plate	ME, EG, DS	ST/FA^j^
“Rehabilitation Exercise System” [10]	—	—	—	Pressure mat	—	ST/FA
Scoliosis Apps [12]	—	—	—	—	ScolioTrack, ScolioMeter, APECS^k^, CobbMeter, ScolioDetector, Scoliosis Tracker, Scoliosis Manager (web) or functions: measurement of spinal curvature (ATR^l^ or Cobb angle), posture tracking, exercise instructions, remote monitoring, documentation, compliance tracking/smartphone hardware used: IMU and camera	—

a BPD: body pressure distribution.

b IMU: inertial measurement unit.

c ME: monitoring of exercise execution.

d EG: exercise guidance.

e DS: data storage.

f FT/IE: free training, including training equipment.

g Not applicable.

h FT/EE: free training, excluding training equipment.

i GF: gamification.

j ST/FA: stationary training on fixed apparatus.

k APECS: Artificial Intelligence Posture Evaluation and Correction System.

l ATR: angle of trunk rotation.

### Motion Tracking and Respiration Tracking

Motion tracking is one of our core components for monitoring exercise execution. To achieve this, we have integrated 9 inertial sensors into a textile wearable, which also allow for monitoring of posture. During the technical verification of our motion tracking, we examined 3 representative samples of the 9 iScoolio boards. Compared to the optical reference system (ARAMIS 3D), this resulted in an average absolute deviation of 2.29° (SD 1.66°), supporting H_1_. It should be noted that the significant outliers in the reference system contributed to an increase in the deviation. These are most likely due to delays or errors in manual image capture. Similar deviations between IMU in wearable applications and an optical system (OptiTrack) were found, for example, by Li et al [16] with 2.00° (SD 2.65°), Teufl et al [17] with ≤2.40°, and Bell et al [18] with ≤3.00°. In terms of repeatability, the iScoolio boards achieved good results with a maximum SD of 1.48°. Studies by Fennema et al [19], Hamidon et al [20], and Beange et al [21] also show that IMUs can exhibit high repeatability and be suitable for motion tracking concepts. We have thus created a basis for monitoring scoliosis exercises.

Unlike the wearables for home-based scoliosis training described in the literature, we have integrated breathing tracking into our system. In many Schroth exercises, the number of breaths taken is used as a counting variable for repetitions. Common measurement systems for respiration that can also be worn on the body include inertial sensors [22,23], stretch sensors [23-26], and bioimpedance sensors [23,27]. Due to their good integrability, we opted for textile strain sensors. Although this measurement principle is susceptible to motion artifacts [26], it is nevertheless very well suited to our application due to the classic sequence of many Schroth exercises: (1) adopting a corrective posture and (2) targeted breathing.

For the technical verification of the sensor concept, we examined 2 respiratory parameters relevant to scoliosis therapy: respiratory rate and respiratory depth. Both can be extracted from the raw data measured using the strain sensors we integrated. This enabled us to verify H_2_. However, automated evaluation requires the integration of additional data processing. Low-pass filters and finite impulse response filters, as well as window size [28], maximum-minimum, zero-crossing, and threshold detection algorithms [29], can be used to detect respiratory rate. For the detection of breathing depth, it is sufficient to differentiate between the measured values, as the increasing filling volume of the lungs is accompanied by an increasing expansion of the sensor.

By supplementing motion tracking and posture monitoring with components for measuring respiratory parameters, we have created a technical basis for recording RAB. Further software development and the integration of machine learning approaches are required for accurate tracking.

### Force Tracking Upper Limbs

To provide the most versatile workout possible, we have combined the features of wall bars and exercise sticks into a single training device, the Bars-Module. The supporting feet and handles of this module can be used to measure forces in the upper limbs. The technical verification showed that the demonstrator’s raw sensor signals responded clearly and proportionally to applied forces, which substantiates H_3_. On the basis of the 3 selected force levels, nonlinear behavior was observed when considering the entire measuring range, as well as drift behavior. These characteristics, supplemented by the uninvestigated hysteresis, reflect the underlying resistive measuring principle [30,31]. However, these negative effects can be significantly reduced through the use of appropriate algorithms [31]. In addition to the software options, optimization through hardware adaptation would also be conceivable. Due to the available installation space, the force-sensing resistor sensors in the supporting feet could be replaced by high-quality load cells, which in turn would mean a significantly higher financial outlay.

Thanks to the integrated measurement functions of the Bars-Module, there are various options for monitoring the exercise execution. In addition to traditional grip strength, which correlates with scoliosis [32], it is possible to measure isometric force during an exercise. In combination with the posture tracking feature of the Wearable-Module, it is possible to determine whether the user maintains their corrective posture throughout a set. Furthermore, the ability to distinguish between the left and right arms allows for the determination of symmetries or asymmetries in force distribution. In combination with the other modules, this enables an analysis of how the user assumes their corrective posture.

### Body Pressure Distribution

To further increase the variety of exercises, we have added a second training device to our concept. We chose a balance board, which plays an important role in many PSSE exercises. The basis for this is the impairment of the cortical centers of the brain in scoliosis, which are responsible for controlling balance [2]. In their study involving 195 participants (adolescent idiopathic scoliosis [AIS]: n=114, 58.5%; control: n=81, 41.5%), Le Berre et al were able to show that dynamic proprioception is selectively altered in AIS [33]. Targeted training of balance reactions is therefore intended to improve axial, static, and dynamic balance, which in turn helps to intensify self-correction during everyday activities [2]. As an extension of the training functions of conventional balance boards, we have also equipped our module with measuring functions. Integrated sensors can be used to determine both the inclination of the board and the pressure distribution on the standing surface. This makes it possible to monitor the execution of training instructions. We have also created the hardware basis for investigating parameters such as foot pressure distribution and COP, both of which are influenced by scoliosis [34-37]. In their study, Horng et al found a statistically significant linear relationship between the Cobb angle and maximum foot pressure on the hallux [35]. Regarding COP, the study by Larni et al showed a decline in COP parameters in adolescents with AIS compared to the healthy control group in tests using a Kistler force plate [37]. In addition to these standing measurement applications, balance boards equipped with sensors also offer various application possibilities when sitting. Jung et al were able to use integrated acceleration sensors to measure the asymmetrical sitting postures caused by scoliosis and pelvic deformity in patients with AIS [38]. To record all these parameters in detail, further development steps are necessary for our system.

Regarding the detection of stance width, our measurements showed slight absolute deviations from the mean, with a maximum of 1.2 cm. Comparing this value with the intervals described in the literature for targeted changes in the training effect, such as a narrow stance of 31.7 (SD 2.7) cm and a wide stance of 59.8 (SD 5.1) cm [39], it can be seen that these variations are sufficiently small and therefore have no significant negative effect on the measurements.

The verification of our COP measurement function showed that 48% of the measurements had a deviation of ≤1 box and 82% had a deviation of ≤2 boxes. With a sensor element resolution of 10 mm, 82% of the measured values had a COP shift of ≤20 mm. It should also be noted that a single sensor element has a diameter of 6 mm. With a center-to-center distance of 10 mm between these sensor elements, this results in horizontal and vertical dead zones of 4 mm and diagonal dead zones of 8.14 mm. Depending on the positioning of the test weights, this may have resulted in real pressure differences between the 2 clusters, which would also cause a real shift in the COP. The increased edge errors may be attributed to material transitions, higher conductor resistances, or calibration limitations.

These measurement functions allow the user a specific stance width to be set and tracked, which affects the difficulty level of the balance exercises. In addition, the COP can be measured during static standing or targeted balance training. Finally, during seated exercises in which the Balance-Module is used as a support surface, it is possible to measure how hip displacement and seat pressure distribution are used during the exercise.

### App or Web Interface

The Interface-Module completes our system with a digital human-technology interface. On the one hand, it serves to communicate, bundle, and process all sensor data. On the other hand, it contains an exercise catalog and outputs visualizations of the measurement data and therapy instructions via an associated web application. In the targeted therapeutic setting, it functions as a scoliosis-specific software tool, which is represented in the literature with various application scenarios.

Compared to the scoliosis software solutions described by Bottino et al [12], the Interface-Module focuses more on the processing and visualization of sensor data as a basis for exercise monitoring and guidance. Nevertheless, there are minor similarities in terms of the selection of suitable exercises or the provision of information for compliance.

### Limitations

In its current state of development, the iScoolio concept still has some room for improvement. One of the biggest limitations is that motion tracking is currently limited to the torso and head and that all sensor data are recorded at a frequency of only 4 Hz. Furthermore, all iScoolio boards are independent components, which therefore require a separate charging and calibration process. This results in a time-consuming setup process. In addition, exercises in the supine position are not possible due to the size of the current boards. For the training modules, the aim should be to reduce the size of the Balance-Module. In addition, different force sensors, a faster extension mechanism with less backlash, and a locking function for the supporting feet would be useful for the Bars-Module. All these questions will be addressed in further work.

There were also some limitations regarding technical verification. These included the fact that all measurements were limited to repetitions of a specific Schroth exercise.

For the motion tracking investigation, we limited ourselves to 3 representative iScoolio boards. In addition, only points and angles on the frontal and sagittal planes were examined; mixed movements were not. Nevertheless, these data enable us to draw conclusions about the accuracy of the measurement system, which was the aim of the investigation. Another limitation regarding motion tracking resulted from the manual triggering of the ARAMIS 3D system.

Regarding respiration tracking, the test was likewise limited to a single representative sensor. In addition, the filling and deflation speed of the air cushions was limited by the valve installed in the test setup. Furthermore, the rising and falling of the chest was simulated by 2 air cushions, which only produced a selective increase in volume rather than a full-surface increase. Finally, it should be noted that no classic reference measurement system was used for this measurement, but rather an actuator-based approach with high repeatability.

When verifying the handpieces of the Bars-Module, the greatest limitations were caused by the 3D-printed half-shells. These only represent a hand in a highly simplified form and had inaccuracies due to additive manufacturing. Furthermore, the handles were verified with a round wooden profile dummy instead of the metal rod. Both limitations led to a deviation from the real load but represented a suitable approach for integrating a testing machine with regard to the measurement function to be tested.

In the case of the Balance-Module, the limiting factors during verification were, on the one hand, the test weights, which did not accurately represent a human foot in terms of their shape and weight. However, the smaller outline of the weights enabled a more detailed examination of the sensor surface.

Another limitation is that verification was only carried out in a technical setting and without the involvement of human participants. Therefore, no clinical conclusions can be drawn regarding the impact on the effects of scoliosis, such as pain, reduced quality of life, or depression [40,41].

### Conclusions

In this paper, we have presented the iScoolio concept, which has the potential to support home-based scoliosis therapy through adaptable devices and tracking elements. Integrated sensors enable monitoring of movements, breathing, forces, and pressure distribution during training. An associated web application visualizes the recorded data and serves as a guide for performing the exercises. Using the new approach could increase motivation, reduce concerns about incorrect exercise execution, and enable adherence monitoring and teletherapy. Technical verification indicates that the integrated tracking methods are functional; however, there is still room for optimization in terms of hardware and software. The system’s greatest areas for improvement include the low sampling frequency (4 Hz), the lack of motion tracking for arms and legs, the long setup process, and measurement inaccuracies in some sensor designs. Future work will address these limitations. Before conducting clinical trials under real-world conditions, further validation is required.

## Supplementary material

10.2196/93721 Multimedia Appendix 1 Schroth body blocks.

10.2196/93721 Multimedia Appendix 2 Schroth exercises.

10.2196/93721 Multimedia Appendix 3 Characteristics of motion tracking analysis.

## Abbreviations

AIS: adolescent idiopathic scoliosis
COP: center of pressure
DPU: data processing unit
IMU: inertial measurement unit
PSSE: physiotherapeutic scoliosis-specific exercises
RAB: rotational angular breathing
