# Supplementary material for: Digitally Assisted Training System for Home-Based Scoliosis Therapy: Proof-of-Concept and Technical Verification Study
Source: JMIR Rehabil Assist Technol. 2026 Oct 29;13:e93721. doi: 10.2196/93721 (PMC13718385; doi:10.2196/93721)
Supplement: Supplementary file 1 [file rehab-v13-e93721-s001.pdf]

1. Schroth body blocks

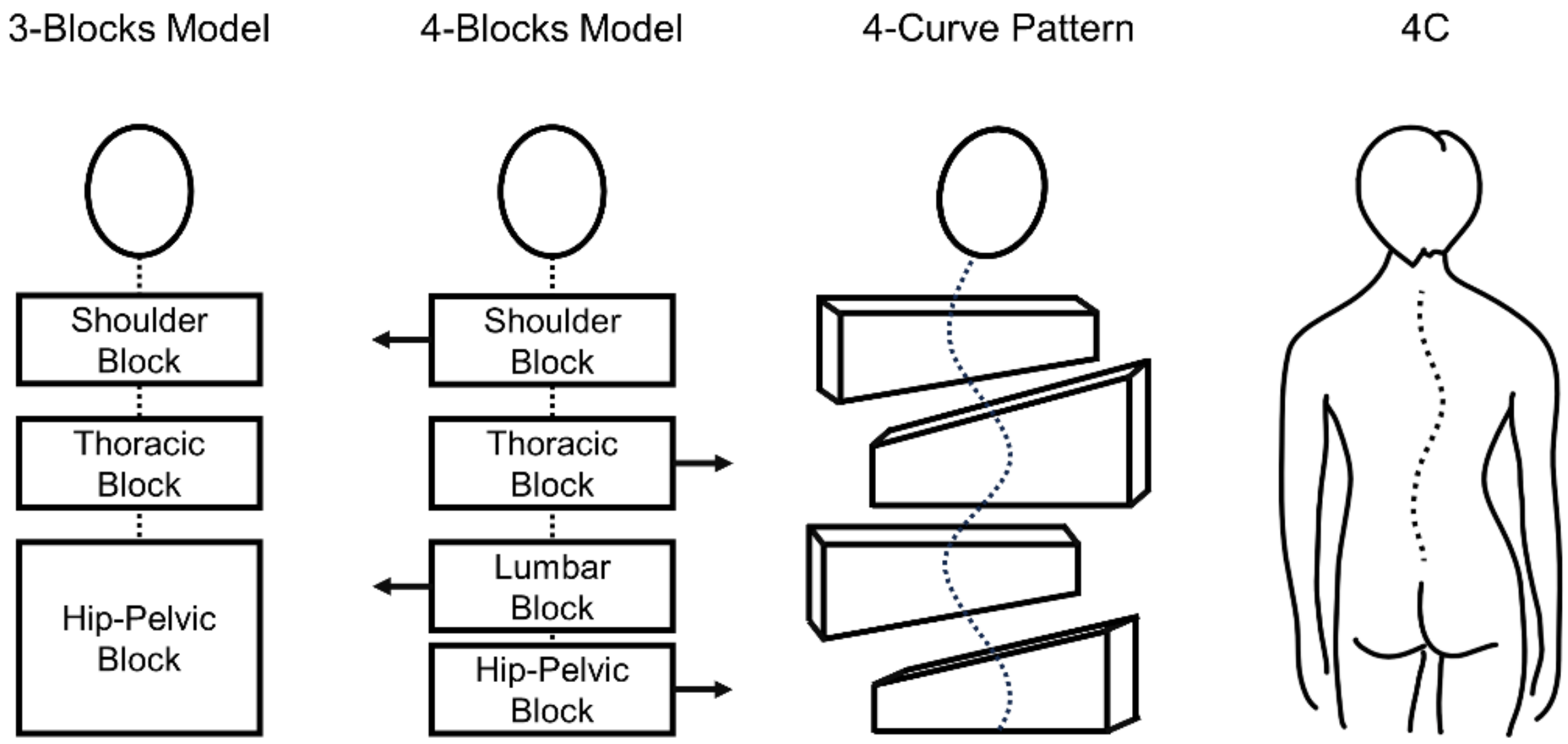

Figure 1: *Block model based on Lehnert-Schroth [1]*

Reference

1. Lehnert-Schroth C, Auner-Gröbl P, editors. Dreidimensionale Skoliosebehandlung: Atmungs-Orthopädie System Schroth : ein physiotherapeutisches Konzept zur Verbesserung von Wirbelsäulenverkrümmungen. 9. Auflage. München: Elsevier; 2021. ISBN:978-3-437-46462-1.
