# Supplementary material for: Digitally Assisted Training System for Home-Based Scoliosis Therapy: Proof-of-Concept and Technical Verification Study
Source: JMIR Rehabil Assist Technol. 2026 Oct 29;13:e93721. doi: 10.2196/93721 (PMC13718385; doi:10.2196/93721)
Supplement: Supplementary file 2 [file rehab-v13-e93721-s002.pdf]

2. Schroth exercises

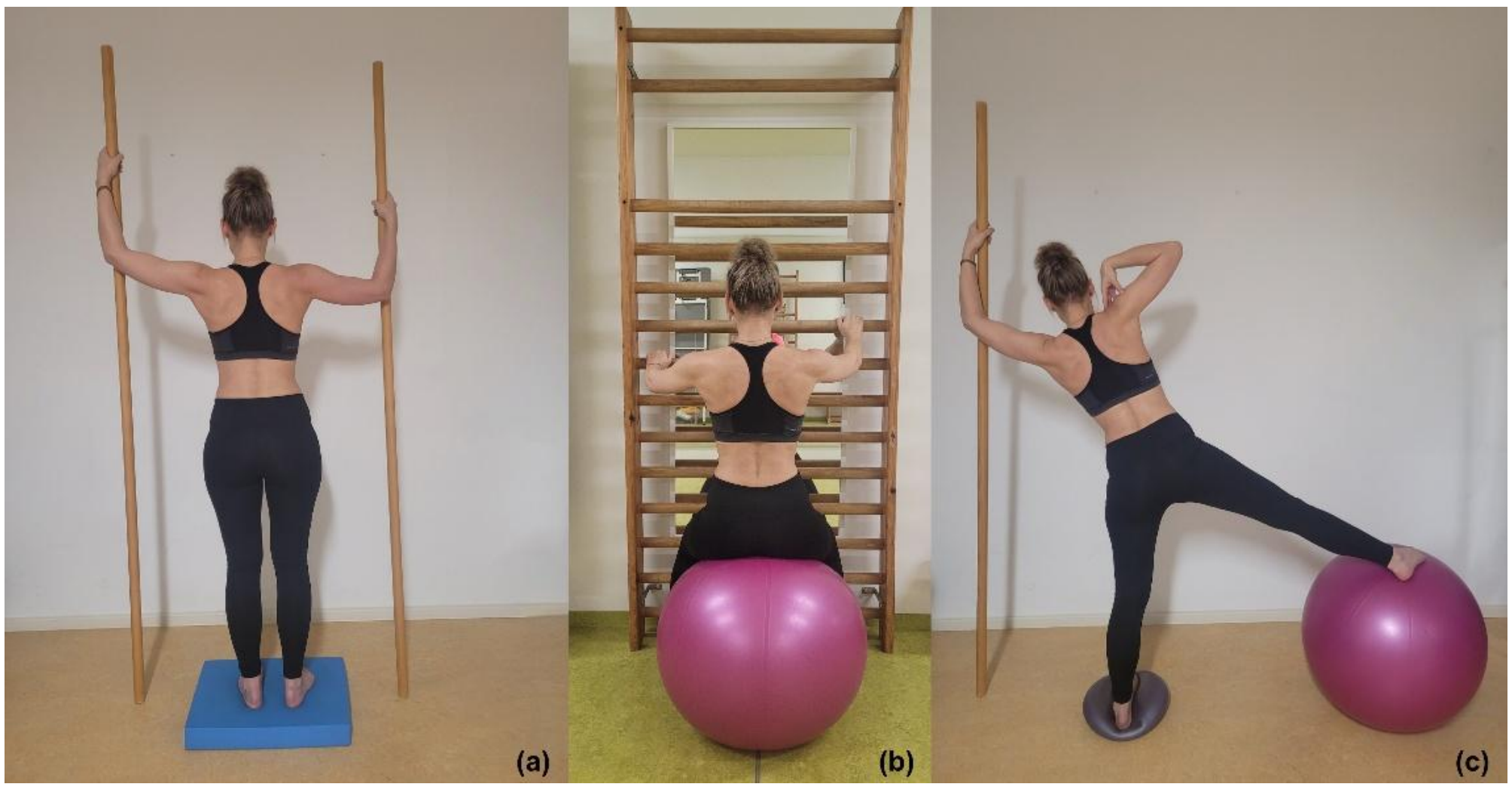

Figure 1: *Typical exercises from Schroth therapy: (a) "Between Two Poles", (b) "50X" and (c) "Sail"*

Between Two Poles

In the "Between Two Poles" exercise, the patient stands on the floor or a balance element (e.g., balance board) and actively corrects their posture. Two vertically aligned bars on the left and right sides of the body serve as supports for assuming and maintaining the corrected position. Targeted breathing in this position then allows shortened areas of the chest to expand and collapsed lung lobes to open. The "Between Two Poles" exercise is therefore considered a very effective stretching exercise for the "thoracic block." [1] The Schroth training script provides the following instructions for this exercise [2]:

- Stand between two bars placed at the height of your toes and assume your corrective posture from caudal to cranial.
- Depending on the therapy plan, the arms are placed in a U-hold or offset position.
- Hold the final position with corrective breathing and muscle tension over several breathing cycles without losing the correction.
- Use the bars for better stabilization by applying slight isometric pressure in the caudal direction.
- Repetitions: 5x3 breaths (beginners), 5x5 breaths (advanced)
- Pause: 30 seconds

50X

In the "50X" exercise, the patient sits on a stool or Pezzi ball in front of a wall bar and performs active 3D self-correction. The rungs, which are gripped with both hands, serve as support for the alignment of the torso. The aim is to push the concave areas "outward and backward" and the convex areas "forward and inward" [1]. To consolidate the corrected position motorically, the change between the starting and end positions should

be repeated at least 50 times, which is the basis for the name of the exercise [3]. The following training instructions are provided for "50X" [2]:

- Sit on a stool or Pezzi ball with your legs stretched out horizontally in front of a wall bar.
- Adjust your corrective posture from caudal to cranial.
- Then perform a stabilization phase (muscle tension) with corrective breathing and gentle symmetrical traction on the rung(s) in the caudal direction.
- Repetitions: 4x6 breaths (beginners), 5x8 breaths (advanced)
- Pause: At least 30 seconds in a conscious everyday posture with arms down

Sail

The "Sail" exercise is used to correct thoracic blockage in the frontal plane and stretch the rib cage. The patient stands with the leg on the L-side (lumbar mountain and/or rib cage) on the floor or on a balance training device. The other leg is stretched out on a stool or Pezzi ball, and the upper body is tilted in line with this. The torso is shifted to the L-side. The hand on the L-side holds a bar in a diagonal arm position with the elbow bent. The following training steps are performed from this starting position [2]:

- Assume the corrective posture while standing on one leg.
- Stabilize the final position you have achieved with corrective breathing.
- Repetitions: 4x6 breaths (beginners), 5x8 (advanced)
- Pause: At least 30 seconds in a conscious everyday posture

References

1. Berdishevsky H, Lebel VA, Bettany-Saltikov J, Rigo M, Lebel A, Hennes A, Romano M, Białek M, M'hango A, Betts T, Mauroy JC de, Durmala J. Physiotherapy scoliosis-specific exercises - a comprehensive review of seven major schools. Scoliosis Spinal Disord 2016;11:20. PMID:27525315
2. Roevenich U. Schulungsskript zur Fortbildung Schroth-Therapeut. [unpublished]. Bad Sobernheim; 2023.
3. Lehnert-Schroth C, Auner-Gröbl P, editors. Dreidimensionale Skoliosebehandlung: Atmungs-Orthopädie System Schroth : ein physiotherapeutisches Konzept zur Verbesserung von Wirbelsäulenverkrümmungen. 9. Auflage. München: Elsevier; 2021. ISBN:978-3-437-46462-1.
