# Supplementary material for: Digitally Assisted Training System for Home-Based Scoliosis Therapy: Proof-of-Concept and Technical Verification Study
Source: JMIR Rehabil Assist Technol. 2026 Oct 29;13:e93721. doi: 10.2196/93721 (PMC13718385; doi:10.2196/93721)
Supplement: Supplementary file 3 [file rehab-v13-e93721-s003.pdf]

3. Characteristics of motion tracking analysis

Table 1: *Wearable-Module's motion tracking measurement data*

	Frontal						Sagittal					
	S3			S5			S3			S5		
	left	right	2 x zero	left	right	2 x zero	anterior	posterior	2 x zero	anterior	posterior	2 x zero
Mean iScoolio	34,29	-31,93	1,584	48,62	-41,89	4,07	30,17	-37,83	-2,922	44,3	-45,76	-1,291
Mean Aramis 3D	32,72	-32,56	0,91	45,31	-43,75	1,265	29,98	-32,84	-0,355	42,56	-41,77	0,025
Deviation mean absolut	1,57	0,63	0,674	3,31	1,86	2,805	0,19	4,99	2,567	1,74	3,99	1,316
Deviation mean relativ	4,58%	1,97%	42,55%	6,81%	4,44%	68,92%	0,63%	13,19%	87,85%	3,93%	8,72%	101,94%
SD iScoolio	0,49	0,63	0,47	0,33	0,45	0,57	1,14	0,46	0,66	1,48	0,54	0,4
SD Aramis 3D	2,11	0,61	0,67	2,52	0,71	1	2,14	1,38	0,55	2,59	1,46	0,34
